# Zinc restores functionality in porcine prepubertal Sertoli cells exposed to subtoxic cadmium concentration *via* regulating the Nrf2 signaling pathway

**DOI:** 10.3389/fendo.2023.962519

**Published:** 2023-02-10

**Authors:** Francesca Mancuso, Iva Arato, Catia Bellucci, Cinzia Lilli, Elena Eugeni, Maria Chiara Aglietti, Anna Maria Stabile, Alessandra Pistilli, Stefano Brancorsini, Francesco Gaggia, Mario Calvitti, Tiziano Baroni, Giovanni Luca

**Affiliations:** ^1^ Department of Medicine and Surgery, University of Perugia, Perugia, Italy; ^2^ Division of Medical Andrology and Endocrinology of Reproduction, Saint Mary Hospital, Terni, Italy; ^3^ International Biotechnological Center for Endocrine, Metabolic and Embryo-Reproductive Translational Research (CIRTEMER), Department of Medicine and Surgery, University of Perugia, Perugia, Italy

**Keywords:** Sertoli cells, cadmium, zinc, oxidative stress, Nrf2 signaling pathway

## Abstract

**Introduction:**

Among substances released into the environment by anthropogenic activities, the heavy metal cadmium (Cd) is known to induce severe testicular injury causing male subfertility/infertility. Zinc (Zn) is another heavy metal that, unlike Cd, is physiologically present in the testis, being essential for spermatogenesis. We aimed to examine the possibility that 50 µM ZnCl_2_ could counteract the toxic effects induced by Cd in an *in vitro* model of porcine prepubertal Sertoli cells (SCs) exposed to both subtoxic (5 μM) and toxic (10 μM) concentrations of CdCl_2_ for 48 h.

**Materials and Methods:**

Apoptosis, cell cycle, and cell functionality were assessed. The gene expression of Nrf2 and its downstream antioxidant enzymes, ERK1/2, and AKT kinase signaling pathways were evaluated.

**Materials and Results:**

We found that Zn, in co-treatment with subtoxic and toxic Cd concentration, increased the number of metabolically active SCs compared to Cd exposure alone but restored SC functionality only in co-treatment with subtoxic Cd concentration with respect to subtoxic Cd alone. Exposure of Cd disrupted cell cycle in SCs, and Zn co-treatment was not able to counteract this effect. Cd alone induced SC death through apoptosis and necrosis in a dose-dependent manner, and co-treatment with Zn increased the pro-apoptotic effect of Cd. Subtoxic and toxic Cd exposures activated the Nrf2 signaling pathway by increasing gene expression of Nrf2 and its downstream genes (SOD, HO-1, and GSHPx). Zn co-treatment with subtoxic Cd attenuated upregulation on the Nrf2 system, while with toxic Cd, the effect was more erratic. Studying ERK1/2 and AKT pathways as a target, we found that the phosphorylation ratio of p-ERK1/2 and p-AKT was upregulated by both subtoxic and toxic Cd exposure alone and in co-treatment with Zn.

**Discussion:**

Our results suggest that Zn could counteract Cd effects by increasing the number of metabolically active SCs, fully or partially restoring their functionality by modulating Nrf2, ERK1/2, and AKT pathways. Our SC model could be useful to study the effects of early Cd exposure on immature testis, evaluating the possible protective effects of Zn.

## 1 Introduction

Infertility is a universal health problem with a detrimental impact on the lives of many couples to whom it can cause severe psychological distress (1). It has been estimated that 8% to 12% of couples in the world are infertile; therefore, 48 million couples and 186 million individuals live in a condition of infertility (2).

In Europe and the United States, it is estimated that 15% of couples and 7.5% of men are infertile (3), with a male factor being the primary or contributing cause in approximately 50% of couples (4).

Male infertility can be caused by numerous factors, which can alter the number or quality of spermatozoa or block their proper release with ejaculation. Many known congenital or acquired causes can impair seminal parameters, but a large percentage of cases, between 30% and 50% (5), remain of unknown origin. Such cases include idiopathic male infertility, in which the seminal fluid analysis shows alterations for which no clear etiology is recognized, or unexplained male infertility, in which the seminal fluid appears normal, and the female partner has no recognized fertility problems (6).

Given the prevalence and relevance of male infertility, great emphasis has been given to the search for potential risk factors for idiopathic infertility or concomitant factors that may provoke or worsen the condition.

Occupational or environmental exposure to some chemical pollutants and related oxidative stress have been recognized to be very important risk factors (7, 8).

Male subjects are exposed to environmental pollutants throughout their life cycle, including the embryonic and fetal period, when exposure to toxic compounds may cause subfertility/infertility later in adulthood (9, 10). Exposure during childhood may be particularly harmful, as it could damage spermatogonial stem cells, depleting the spermatogonial pool with a limited chance of future recovery (11).

Many different pollutants have been linked to testicular damage, some of which may act by mimicking certain endogenous hormones, thus interfering with normal endocrine processes.

These so-called endocrine-disrupting compounds (EDCs) include heavy metals (12–14). Cadmium (Cd) is a heavy metal found in most human foods, which are the main source of exposure in the non-smoking population (15–17).

Cigarette smoke and polluted water and air are additional sources of Cd, which is also found as an environmental pollutant, produced as a result of processes in the metallurgical and mining industries, the production of plastic stabilizers and batteries, and other industrial activities. Although exposure to Cd is low, its accumulation in the human body and its elimination half-life of 20–40 years cause damage to various organs (18). Cd accumulates in the testis where it causes lesions and impairs spermatogenesis.

In the adult testis, Sertoli cells (SCs) play an important role, providing physical support to germ cells and creating a metabolic and immunological environment suitable for spermatogenesis (19, 20).

In humans, SCs proliferate greatly during the fetal period and then slow down during the prepubertal phase until reaching the adult level at puberty (21, 22).

During the fetal and neonatal period, SCs play a critical role in the assembly of testis cords (23), in the organization of the microenvironmental niche in which germ cell differentiation into spermatogonia occurs until the first postnatal month, and in the secretion of anti-Müllerian hormone (AMH), which causes regression of Müller’s duct (24). In the prepubertal period, SCs produce a high level of AMH, being the only active testicular cells. Incidentally, the exact function of AMH in spermatogenesis is still not entirely clear, and it could represent a possible marker of SC function in humans and other prepubertal mammals (25).

Given their important role since the fetal period, it is understandable how early SCs damage can lead to adult male infertility.

In the fetal and neonatal stage, Cd has already been shown to negatively affect the development of immature SCs in the piglet testis, inhibiting their proliferation and causing apoptosis and DNA damage (26). In addition, Cd inhibits the interaction between neonatal SCs and gonocyte in primary murine SC-gonocyte cocultures (27).

In our previous work, we tested the effect of different pollutants on an *in vitro* model of prepubertal porcine SC cultures (28, 29) and verified that Cd causes dysfunction in SCs (30).

Another heavy metal, zinc (Zn), unlike cadmium, has a physiological function in the body, since it is a trace element necessary for the functioning of more than 300 enzymes. Zn is normally found in the testis and is essential in the physiological reproductive process as a necessary cofactor for metalloenzymes involved in spermatogenesis (31), to such an extent that its deficiency in man could lead to production of poorly functional spermatozoa and male infertility (32). Previous studies have shown that Zn supplementation can mitigate testicular damage due to metals such as Pb (33) and improve sperm quality in infertile men (34).

It is known that Cd competes with Zn and displaces it from its normal localization, thus altering Zn physiological function (35) and that Zn administration provides protection against Cd-induced testicular toxicity in adult rat testes (36).

However, there are no studies in the literature explaining the protective mechanism of Zn, even though Zn-containing supplements are routinely administered to patients with idiopathic infertility in clinical practice (37).

Given that primary cell cultures are considered the “gold standard” of *in vitro* models, and they are able to mimic more closely the real characteristics of target tissues *in vivo* (38), in the present paper, we used our already proven primary culture model of porcine prepubertal SCs, an experimental animal model exhibiting significant physiological similarity with humans as further supported by literature data (39), which performed integrated analysis of testicular cells in both pigs and humans. Results of cell-quality metrics demonstrated that human transcriptome data were comparable to those of pigs. In addition, the DEGs and GO analysis performed on each cell type revealed that pig and human biological processes are comparable (39).

The aim of the present study was to investigate whether the damage suffered by prepubertal pig SCs exposed to Cd in terms of cell viability and function could be restored or attenuated by co-treatment with Zn and then investigate the mechanisms underlying these possible effects.

## 2 Materials and methods

### 2.1 SCs isolation and characterization

Animal studies were conducted in agreement with the guidelines adopted by the Italian Approved Animal Welfare Assurance (A- 3143-01) and the European Communities Council Directive of 24 November 1986 (86/609/EEC). The experimental protocols were approved by the University of Perugia. Danish Duroc neonatal pigs (15 to 20 days old) underwent bilateral orchidectomy after general anesthesia with ketamine (Ketavet 100; Intervet, Milan, Italy), at a dose of 40 mg/kg, and dexmedetomidine (Dexdomitor, Orion Corporation, Finland), at a dose of 40 g/kg, and were used as SC donors. Specifically, pure porcine neonatal SCs were isolated and characterized, according to previously established methods (30). Briefly, the fibrous capsule was removed. Then, the testes were finely chopped and digested twice enzymatically, with a mixed solution of trypsin and deoxyribonuclease I (DNase I) in Hanks’ balanced salt solution (HBSS; Merck KGaA, Darmstadt, Germany) and collagenase P (Roche Diagnostics S.p.A., Monza, Italy). The tissue pellet was centrifuged, passed through a 500-μm-pore stainless steel mesh and then resuspended in glycine to eliminate residual Leydig and peritubular cells. The pellet was then collected and kept in HAM’s F12 medium (Euroclone, Milan, Italy) and added with 0.166 nmol/L retinoic acid (Sigma-Aldrich Co., St. Louis, MO, USA) and 5 ml per 500 ml of insulin-transferrin-selenium (ITS, Becton Dickinson cat. no. 354352; Franklin Lakes, NJ, USA) in 95% air/5% CO_2_ at 37°C. Cells were cultured for 3 days. To detect the presence of AMH and vimentin (SCs markers), 3β-Hydroxysteroid dehydrogenase (3β HSD) (Leydig cell marker), and alpha smooth muscle actin (ASMA) (peritubular cell marker), immunostainings were performed according to previously reported methods (40, 41). Briefly, untreated and treated SC monolayers were grown on glass chamber slides (LabTek II, Nunc, Thermo Fisher, Rochester, NY, USA), and fixed in ice-cold methanol for 15 min. The fixed cells were then permeabilized with 0.2% Triton X-100 (Sigma-Aldrich Co., St. Louis, MO, USA) in PBS (Euroclone, Milan, Italy) for 10 min at room temperature and blocked with 0.5% BSA (Sigma-Aldrich Co., St. Louis, MO, USA) in PBS for 1 h prior to exposure to AMH primary antibody (sc-6886; polyclonal goat anti-rat, 1:100, Santa Cruz Biotechnology, Temecula, CA, USA), to INSL-3 (sc-515120, monoclonal mouse anti-human, 1:200, Santa Cruz Biotechnology, Temecula, CA, USA), to ASMA (ab5694; polyclonal rabbit anti-pig, 1:200, ABCAM, Cambridge, UK), and to vimentin (ab8978; monoclonal mouse anti-pig, 1:100 Abcam, Cambridge, UK), overnight at +4°C. The cells were then washed in PBS three times (5 min each time) and then exposed to a secondary Alexa 488-conjugated donkey anti-goat antibody (1:500, Molecular Probes, NY, USA), Alexa 488-conjugated donkey anti-rabbit (1:500, Molecular Probes, NY, USA), and Alexa 488-conjugated donkey anti-mouse (1:500, Molecular Probes, NY, USA). The cells were then treated with RNase (10 mg/ml, Sigma-Aldrich Co., St. Louis, MO, USA) and counterstained for 1 min with DAPI (Sigma-Aldrich Co., St. Louis, MO, USA). Negative controls were included without the primary antibody treatment. The cells were mounted with ProLong^®^ Gold antifade reagent (Molecular Probes, NY, USA). To evaluate the percentage of AMH, vimentin, 3β HSD, and ASMA-positive cells, chamber slides were analyzed using a BX-41 microscope (Olympus, Tokyo, Japan) equipped with a fluorescence photocamera. The isolated SC culture was 95% pure as indicated by immunostaining for AMH and vimentin (**Figures S1A, B**, respectively) with an extremely low percentage of non-SC cells (<5%) characterized by immunostaining for 3β-HSD (Leydig cells, **Figure S1C**) and ASMA (peritubular cells, **Figure S1D**).

### 2.2 SC culture and treatments

SCs were maintained at 37°C in a 5% CO_2_ humidified atmosphere in the absence (unexposed-control group) or presence of 5 or 10 µM CdCl_2_ (Cd) (Sigma Chemical Co, St. Louis, MO) (exposed group) according to our previous article (30) and/or 50 µM ZnCl_2_ (Zn) (Sigma Chemical Co, St. Louis, MO) on the basis of literature data (42) for 48 h in HAMF12 (Euroclone, Milan, Italy) supplemented with 0.166 nM retinoic acid (Sigma-Aldrich Co., St. Louis, MO, USA) and 5 ml/500 ml of Insulin-Transferrin-Selenium (ITS) + Premix (Cat. No. 354352; Corning, MA, USA).

### 2.3 Cytotoxicity assay

In SCs (1 × 10^5^ cells/well) seeded in 96-well plates and exposed to Cd and/or Zn as described earlier, cell viability was assessed by 3-(4,5-dimethylthiazol-2-yl)-2,5-diphenyltetrazolium bromide (MTT test) (29). Data were reported as mean ± SD of eight replicates obtained from four independent experiments.

### 2.4 Apoptosis assessment

The detection of phosphatidylserine externalization was performed using an Annexin V Apoptosis Detection Kit (K101-100 BioVision CA, USA), made up of annexin V–fluorescein isothiocyanate (AnV–FITC) and propidium iodide–phycoerythrin (PI–PE), which are able to differentiate viable from necrotic and apoptotic cells. The aliquots of experimental samples were washed with PBS (Euroclone, Milan, Italy), centrifuged, and suspended in 500 μl of Annexin binding buffer to obtain a cell count of approximately 1 × 10^5^. Five microliters of AnV–FITC and 5 μl of PI–PE (50 μg/ml) were added to each cell suspension. The samples were incubated at RT for 5 min in the dark and then analyzed by a flow cytometer. Flow cytometry analysis was performed by plotting green fluorescence (FL1)/AnV–FITC *vs*. red fluorescence (FL2)/PI–PE positive cells. The combination of AnV and PI allows the discrimination of four cell categories: viable cells (AnV−/PI−), early apoptotic cells (AnV+/PI−), late apoptotic cells (AnV+/PI+), and necrotic cells (AnV−/PI+). The sum of apoptotic cells was also calculated. Flow cytometry data acquisition was performed on a FACSscan Calibur (Becton Dickinson, Milan, Italy) equipped with 488- and 633-nm lasers and running CellQuest Software (Becton Dickinson, CA, USA). Ten thousand events were collected for each sample (43).

### 2.5 Cell cycle

SCs were plated at a density of 3 × 10^6^ cells/well in a six-well plate and incubated for 48 h. After incubation, cells were sequentially trypsinized, washed with PBS, fixed overnight in 70% ice-cold ethanol (Sigma-Aldrich Co., St. Louis, MO, USA), at 4°C, resuspended in PBS plus 30 µg/ml RNase-A (Sigma-Aldrich Co., St. Louis, MO, USA), and incubated at 4°C for 5 min. Following the addition of FACS buffer (PBS + 2% FBS, Euroclone, Milan, Italy) and PI (1 mg/ml; Sigma-Aldrich Co., St. Louis, MO, USA), cells were incubated at 4°C for 30 min and finally DNA content was measured using a FACSCalibur flow cytometer. CellQuest software (Becton Dickinson, CA, USA) was used to quantify the distribution of cells in each cell cycle phase: sub-G_1_ (dead cells), G_0_/G_1_, S, and G_2_/M. Twenty thousand live events were collected for each sample and experiments were performed in triplicate. Apoptotic cells with hypodiploid DNA content were measured by quantifying the sub-G_1_ peak in the cell cycle pattern (43, 44).

### 2.6 ROS expression

Intracellular reactive oxygen species (ROS) were measured *via* flow cytometric analysis by treating 1 × 10^6^ unexposed and exposed SCs with 1 μM dichlorofluorescein diacetate (DCFH-DA, Sigma-Aldrich Co., St. Louis, MO, USA) solution in PBS at 37°C for 30 min, after supernatant removal and washing with PBS, 500 μl of FACS buffer (45) was added. Fluorescence was read by flow cytometry using a FACSscan Calibur equipped with 488- and 633-nm lasers and running CellQuest software.

The sensitivity of the test was confirmed by adding 30 µM hydrogen peroxide (H_2_O_2_) (30 min) on unexposed SCs as positive control (46).

### 2.7 RNA isolation, reverse transcription, and real-time reverse transcriptase-polymerase chain reaction analyses

Total RNA was isolated with Tri-reagent (Sigma-Aldrich Co., St. Louis, MO, USA) and quantified by reading the optical density at 260 nm. Subsequently, 2.5 μg of total RNA was subjected to reverse transcription (RT Thermo Scientific, Waltham, MA, USA) in a final volume of 20 μl. Reverse transcriptase-polymerase chain reaction (RT-PCR) was performed using 25 ng of the cDNA and SYBR Green master mix (Stratagene, Amsterdam, the Netherlands), as previously described (47).

Gene expression versus β-actin was evaluated by RT-PCR on an MX3000P Real-Time PCR System (Agilent Technology, Milan, Italy). The sequences of the oligonucleotide primers are listed in **Table 1**. The thermal cycling conditions were 1 cycle at 95°C for 5 min, followed by 45 cycles at 95°C for 20 s and 58°C for 30 s. The data required for carrying out a comparative analysis of gene expression were obtained by means of the 2-(DDCT) method.

**Table 1:** Primer sequences for PCR analyses.

Gene	Forward	Reverse
**β-Actin** **AMH** **βB subunit (Inhibin B)** **SOD1** **Nrf2** **GHSPX** **HO-1** **Bcl2** **FSHr** **GDNF** **SCF** **LDH** **ABP** **Transferrin** **Aromatase**	ATGGTGGGTATGGGTCAGAA GCGAACTTAGCGTGGACCTG TGGCTGGAGTGACTGGAT TCGGGAGACCATTCCATCAT TTCACTAAACCCAAGTCCCAGCAT CGAGAAGTGTGAGGTGAATGG CTGGTGATGGCGTCCTTGTA GCCACTTACCTGAATGAC TTTCACAGTCGCCCTCTTTCCC AAAGTCGGGCAGGCGTGTT GAATGACAGCAGTAGCAGTAAT GCACCCTGAATTAGGCACTGATG AATAACTGGCTGGACCAA CAGCAGAACACTGACGGAAA CCGTCTGTGCCGATTCCATC	CTTCTCCATGTCGTCCCAGT CTTGGCAGTTGTTGGCTTGATATG CCGTGTGGAAGGATGAGG ACCTCTGCCCAAGTCATCT AAGCCAAGCAGTGTGTCTCCATA GCGGAGGAAGGCGAAGAG TTGTTGTGCTCAATCTCCTCCT TTCCGTAGAGTTCCACAA TGAGTATAGCAGCCAGAGATGACC AAAGGACAGGTCGTCGTCAAAGG TTCTTCCAGTATAAGGCTCCAA ATAAGCACTGTCCACCACCTGTT GAAGAATATCCCAGGTTGAG CTCCTCAGCAACACAGGTCA GAAGAGGTTGTTAGAGGTGTCCAG

Anti-Müllerian hormone (AMH), nuclear factor erythroid-2 related factor 2 (Nrf2), superoxide dismutase 1(SOD1), heme-oxygenase 1 (HO-1), glutathione peroxidase (GSHPx), follicle-stimulating hormone receptor (FSHr), glial cell line-derived neurotrophic factor (GDNF), stem cell factor (SCF), lactate dehydrogenase (LDH), androgen binding protein (ABP).

### 2.8 Protein extraction and Western blot analysis

Total protein extracts were prepared by lysing the cells in 100 μl of radioimmunoprecipitation assay lysis buffer (RIPA buffer, Santa Cruz Biotechnology Inc., Santa Cruz, CA, USA) as previously described (48).

After centrifuging the mixture at 1,000×*g* (Eppendorf, NY, USA) for 10 min, the supernatant was collected, and total protein content was determined by the Bradford method (49). Sample aliquots were stored at −20°C for Western blot (WB) analysis. The cell extracts were separated by 4%–12% sodium dodecyl sulfate-polyacrylamide gel electrophoresis (SDS-PAGE), and equal amounts of protein (70 μg protein/lane) were run and then blotted on nitrocellulose membranes (Bio-Rad, Hercules, CA, USA). The membranes were incubated overnight in a buffer containing 10 mM Tris (Sigma-Aldrich Co., St. Louis, MO, USA), 0.5 M NaCl (Sigma-Aldrich Co., St. Louis, MO, USA), 1% (v/v) Tween 20 (Sigma-Aldrich Co., St. Louis, MO, USA), rabbit anti-ERK1/2 (1:2000, Millipore, MA, USA), mouse anti-phospho-ERK1/2 (1:100, Millipore; MA, USA), anti-AKT (1:100, Cell Signalling, Danvers, MA, USA), rabbit anti-phospho-AKT (1:1,000, Cell Signalling, Danvers, MA, USA), and rabbit anti-caspase 3 antibody (1:1,000, Cell Signaling, Danvers, MA, USA) primary antibodies.

Primary antibody binding was then detected by incubating membranes for an additional 60 min in a buffer containing horseradish peroxidase-conjugated anti-rabbit (1:5,000, Sigma-Aldrich Co., St. Louis, MO, USA) and/or anti-mouse (1:5,000, Santa Cruz Biotechnology Inc.) IgG secondary antibodies. The bands were detected by enhanced chemiluminescence and acquired by the ChemiDoc Imaging System (Bio-Rad, Hercules, CA, USA).

### 2.9 ELISA

Aliquots of the culture media from all the experimental groups were collected and stored at −20°C for the assessment of AMH (AMH Gen II ELISA, Beckman Coulter; intra-assay CV = 3.89%; inter-assay CV = 5.77%) and inhibin B (Inhibin B Gen II ELISA, Beckman Coulter, Webster, TX, USA; intra-assay CV = 2.81%; inter-assay CV = 4.33%) secretion as previously described (50).

### 2.10 Statistical analysis

Normality analysis was performed by Shapiro–Wilk test, and statistical comparisons were analyzed using one-way ANOVA followed by Tukey’s HSD *post hoc* test (SigmaStat 4.0 software, Systat Software Inc., CA, USA). Values were reported as the means ± SEM of three independent experiments, each performed in triplicate. Differences were considered statistically significant at *p < 0.05, **p < 0.001 compared to unexposed SCs; # *p* < 0.05 and ## *p* < 0.001 with respect to 5 μM Cd; § *p* < 0.05 and §§ *p* < 0.001 with respect to 10 μM Cd.

## 3 Results

### 3.1 Cytotoxicity assay

The MTT assay demonstrated that the cytotoxic effects of 5 μM Cd were abolished and those of 10 μM Cd were markedly attenuated by the co-treatment with Zn.

**Figure 1:**
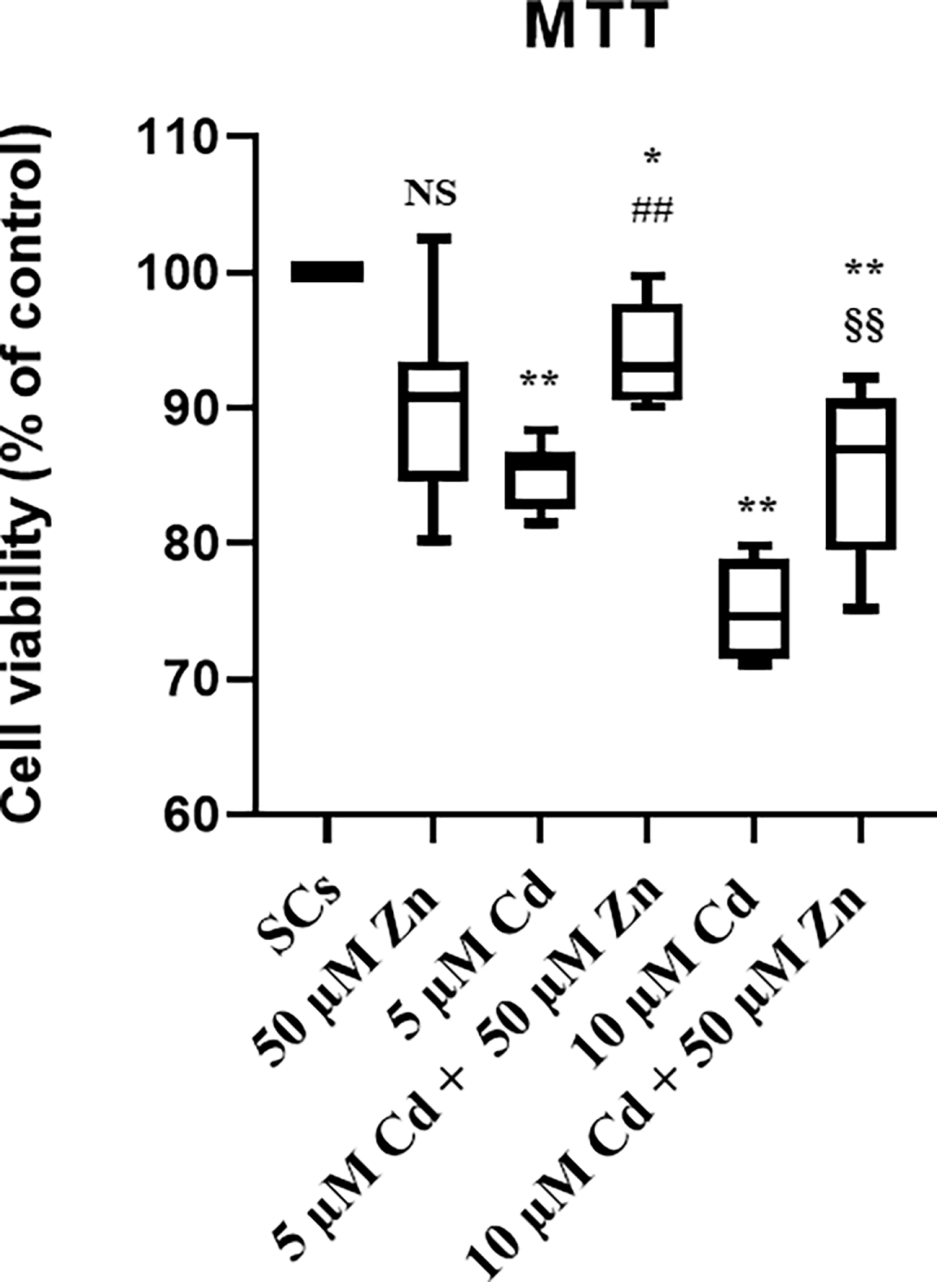
Cell viability by MTT test. Cytotoxicity, measured using MTT reagent in the absence (unexposed-control group, SCs) or presence of 5 or 10 µM CdCl_2_ and/or 50 µM ZnCl_2_ for 48 h. Data presented as the mean ± SEM (NS *vs*. SCs, *p < 0.05 and **p < 0.001 *vs*. SCs, ##*p* < 0.001 *vs*. 5 µM Cd, §§*p* < 0.001 *vs*. 10 µM of three independent experiments, each performed in triplicate).

The cytotoxicity of metal ions was determined by the MTT assay that mainly measures the metabolic activity of cells in culture, indirectly associated with cell viability status. As shown in **Figure 1**, a slight statistically nonsignificant decrease in the percentage of metabolically active cells was observed in SCs exposed to Zn alone compared with unexposed controls. A marked cytotoxic effect was seen in SCs exposed to Cd at both the subtoxic (5 μM) and toxic (10 μM) concentrations, with a significant decrease in the percentage of metabolically active viable cells (**p < 0.001 with −26.2% at 5 μM Cd; **p < 0.001 with −30.1% at 10 μM Cd) compared with unexposed controls.

After co-treatment with 50 μM Zn at both Cd concentrations, the percentages of metabolically active viable SCs increased (##*p* < 0.001 with +21.5% at 5 μM Cd + 50 μM Zn; §§*p* < 0.001 with +18.1% at 10 μM Cd + 50 μM Zn) compared with SCs treated with the corresponding concentration of Cd alone.

### 3.2 Cd and Cd/Zn exposure induced cell cycle disruption and apoptosis in SCs

The results suggested that Cd exposure disrupted the cell cycle in SCs and triggered cycle arrest at the sub-G_1_ phase, apoptosis and necrosis in a dose-dependent manner and co-treatment with Zn was not able to counteract these effects. On the other hand, Zn, the subtoxic Cd and co-treatments triggered an anti-apoptotic response by SCs, in terms of Bcl-2 gene expression compared with unexposed SCs, while toxic Cd showed no response.

To investigate the effects of Cd on cell viability, we analyzed cell cycle progression, apoptosis, and necrosis by flow cytometry and WB analysis.

**Figure 2:**
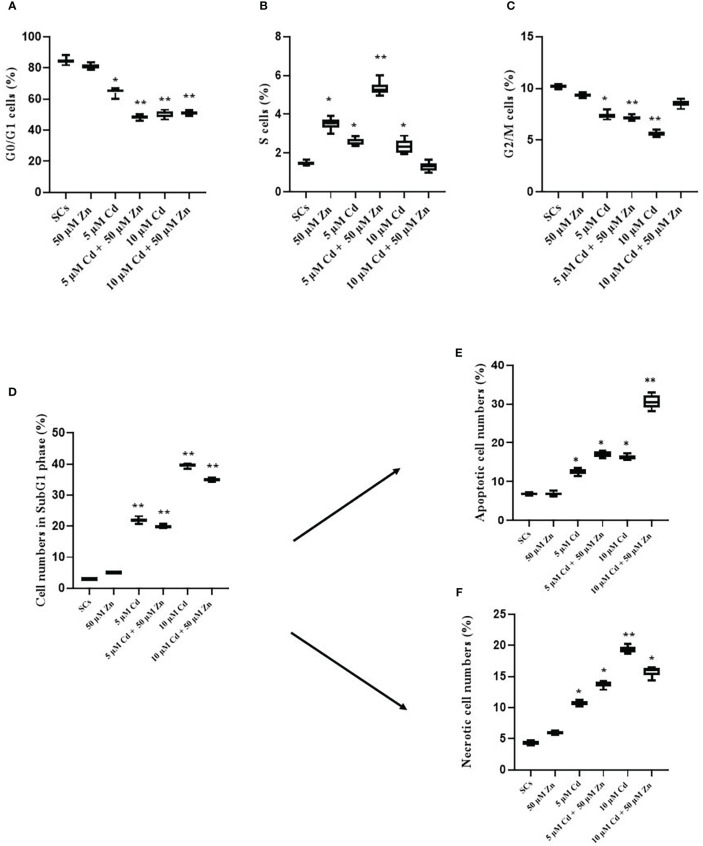
Cell cycle progression, apoptosis, and necrosis. Percentage of cells in G_0_/G_1_ **(A)**, S **(B)**, and G_2_/M **(C)** and sub-G_1_ phase **(D)** in the absence (unexposed-control group, SCs) or presence of 5 or 10 µM CdCl_2_ and/or 50 µM ZnCl_2_ for 48 h. Percentage of apoptotic **(E)** and necrotic **(F)** cells in the sub-G_1_ phase in the absence (unexposed-control group, SCs) or presence of 5 or 10 µM CdCl_2_ and/or 50 µM ZnCl_2_ for 48 h. Data presented as the mean ± SEM (*p < 0.05 and **p < 0.001 *vs*. SCs of three independent experiments, each performed in triplicate).

**Figures 2A–C** show that Cd exposure changed the distribution of SCs in the different cell cycle phases. Indeed, SCs treated with both Cd concentrations significantly accumulated in the sub-G_1_ phase; meanwhile, the proportion of cells in the G_0_/G_1_ phase was markedly decreased, and the proportion of cells in the S phase remained stable.

In particular, 5 μM Cd and 10 μM Cd induced a significant decrease in the percentage of cells in the G_0_/G_1_ phase compared with unexposed SCs (control) (**p < 0.001 with 65.21% and 49.45 *vs*. 84.44%), and the co-treatment with 50 μM Zn induced a significant decrease in the percentage of cells in the G_0_/G_1_ phase compared with control SCs (**p < 0.001 with 49.22 and 51.58% *vs*. 84.44%, respectively, **Figure 2A**).

**Figure 2D** shows the assessment of cells in the sub-G_1_ phase using cell cycle analysis. After 5- and 10-μM Cd exposures, the percentage of cell death was significantly increased in a dose-dependent manner in comparison with unexposed SCs (**p < 0.001 with 22% and 40% *vs*. 3%, respectively).

The co-treatment with 5 μM Cd + 50 μM Zn and 10 μM Cd + 50 μM Zn induced an increase in the percentage of sub-G_1_ cells in comparison with unexposed SCs (**p < 0.001 with 34.34% and 35.49% *vs*. 3.08%, respectively). In contrast, no significant effects were observed after 5 μM Cd + 50 μM Zn and 10 μM Cd + 50 μM Zn with respect to 5 μM Cd and 10 μM Cd, even if a trend downward decrease was observed.

To evaluate whether subtoxic and toxic Cd concentrations induced apoptosis or necrosis in SCs, we used several experimental approaches.

To validate the pro-apoptotic effects of 5 μM Cd and 10 μM Cd in SCs, cells were analyzed by flow cytometry using Annexin V-FITC and results are shown in **Figure 2E**. The percentage of total (early and late) apoptotic cells increased in a dose-dependent manner after Cd treatments. In particular, in the 5 μM Cd and 10 μM Cd exposed SCs, the percentage of apoptotic cells significantly increased compared with unexposed SCs (*p < 0.05 with 12.41%, 16.06% *vs*. 6.5%, respectively).

Zn co-treatment with both Cd concentrations induced a significant increase in the percentage of apoptotic cells compared with unexposed SCs (*p < 0.05 and **p < 0.001 with 17.23% and 31.92% *vs*. 6.5% and 6.75%, respectively).

Necrotic effects of 5 μM Cd and 10 μM Cd in SCs were analyzed by flow cytometry using Annexin V-FITC and results are shown in **Figure 2F**. The percentage of necrotic cells increased in a dose-dependent manner. At 48 h post-treatment, both Cd concentrations significantly increased the percentage of necrotic cells compared with unexposed SCs (*p < 0.05 and **p < 0.001 with 10.79% and 18.64% *vs*. 4.5%, respectively). Zn co-treatment with both Cd concentrations induced a significant increase in the percentage of necrotic cells compared with unexposed SCs (*p < 0.05 with 13.77% and 15.65% *vs*. 4.5%, respectively). In contrast, no significant effects were observed after Zn co-treatment with both Cd concentrations compared with 5 μM and 10 μM Cd alone.

**Figure 3:**
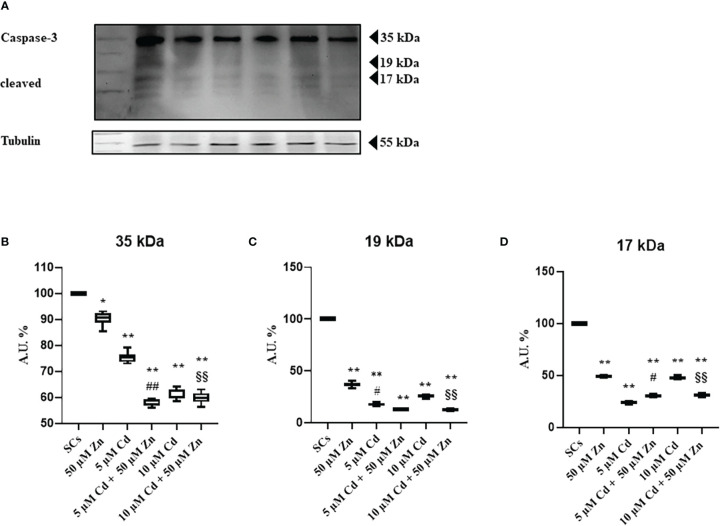
WB analysis of apoptosis. **(A)** Immunoblots of caspase-3 p35, p19, and p17 in the absence (unexposed-control group, SCs) or presence of 5 or 10 μM CdCl_2_ and/or 50 µM ZnCl_2_ for 48 h. Densitometric analysis of the protein bands of caspase-3 p35 **(B)**, p19 **(C)**, and p17 **(D)** in the absence (unexposed-control group, SCs) or presence of 5 or 10 µM CdCl_2_ and/or 50 µM ZnCl_2_ for 48 h. Data presented as the mean ± SEM (*p < 0.05 and **p < 0.001 *vs*. SCs, #*p* < 0.05 and ##*p* < 0.001 *vs*. 5 µM Cd, §§*p* < 0.001 *vs*. 10 μM of three independent experiments, each performed in triplicate).

To further investigate the mechanisms underlying cell death induced by Cd, we assessed, by WB analysis, proteins such as caspase-3, a key enzyme that plays a crucial role in the cell cycle and apoptosis regulation and is partially or totally responsible for the proteolytic cleavage of many key proteins (40). Activation of caspase-3 requires proteolytic processing of its inactive zymogen (p35) into activated p19 and p17 fragments (51), and we found a slight decrease of the inactive form and active fragments after Cd exposures alone or in combination with Zn, compared with untreated SCs (**Figures 3A–D**).

The supposed contradiction between the increase in apoptosis and the decrease in caspase-3 activation could be due to the progress toward the final degradation phase of apoptosis (52).

**Figure 4:**
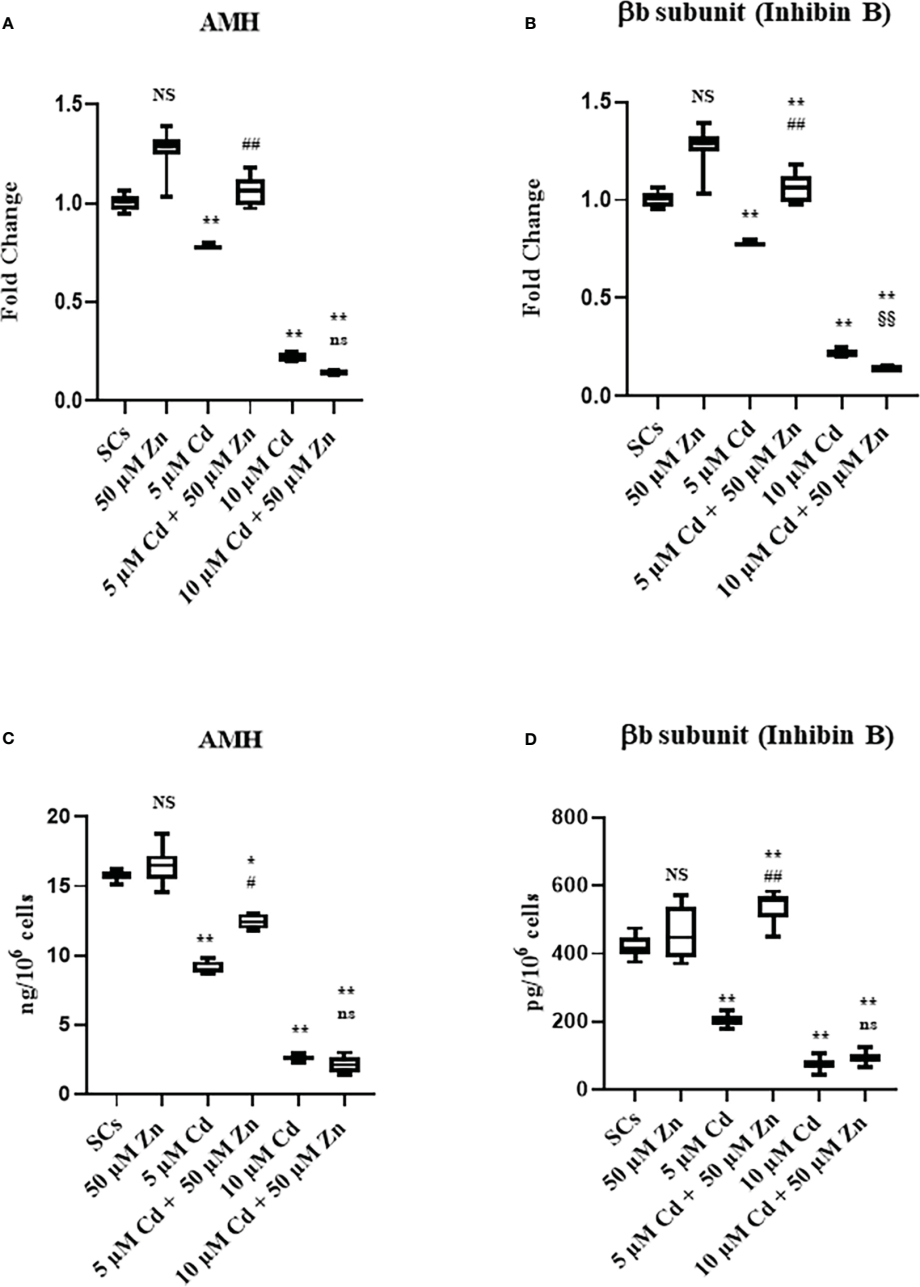
Gene expression and protein secretion of AMH **(A, C)**, and βB subunit (Inhibin B) **(B, D)** in the absence (unexposed-control group, SCs) or presence of 5 or 10 µM CdCl2 and/or 50 µM ZnCl2 for 48 h. Data represent the mean ± S.E.M. (Not significant, NS). NS vs SCs, *p<0.05 and **p<0.001, #p<0.05 and #p<0.05 vs 5 µM Cd, §p<0.05 vs 10 µM, (not significant, ns) ns vs 10 µM of three independent experiments, each performed in triplicate.

To further explore the Cd-induced effects on SC apoptosis, expression of apoptosis-related Bcl-2 gene was detected using real-time PCR, as shown in **Figure 4**. This gene was increased by 310% after Zn alone exposure, and by 320% with 5 μM Cd, whereas 10 μM Cd was not effective.

Co-treatment with 5 μM Cd + 50 μM Zn increased the Bcl-2 gene expression (**p < 0.001) in comparison with unexposed SCs, but decreased it by 48% (##*p* < 0.001) with respect to 5 μM Cd alone.

Co-treatment with 10 μM Cd + 50 μM Zn increased Bcl-2 gene expression by approximately 33% (*p < 0.05), compared with unexposed SCs, and increased Bcl-2 gene expression by approximately 21% (§*p* < 0.05) in comparison with the corresponding concentration of Cd alone.

### 3.3 Analysis of SC functionality

Given that AMH and inhibin B are specific markers of SC functionality, the effects of metal ions alone and in co-treatment were evaluated in SCs by AMH and βB subunit of inhibin B (inhibin B) gene expression and protein secretion assays.

The results suggested that Cd exposure decreased the functionality of SCs in terms of AMH and inhibin B gene expression and protein secretion. Cell functionality, evaluated as protein secretion, could be partially (AMH secretion) or fully (inhibin B secretion) restored after co-treatment with Zn and 5 μM Cd.

**Figure 5:**
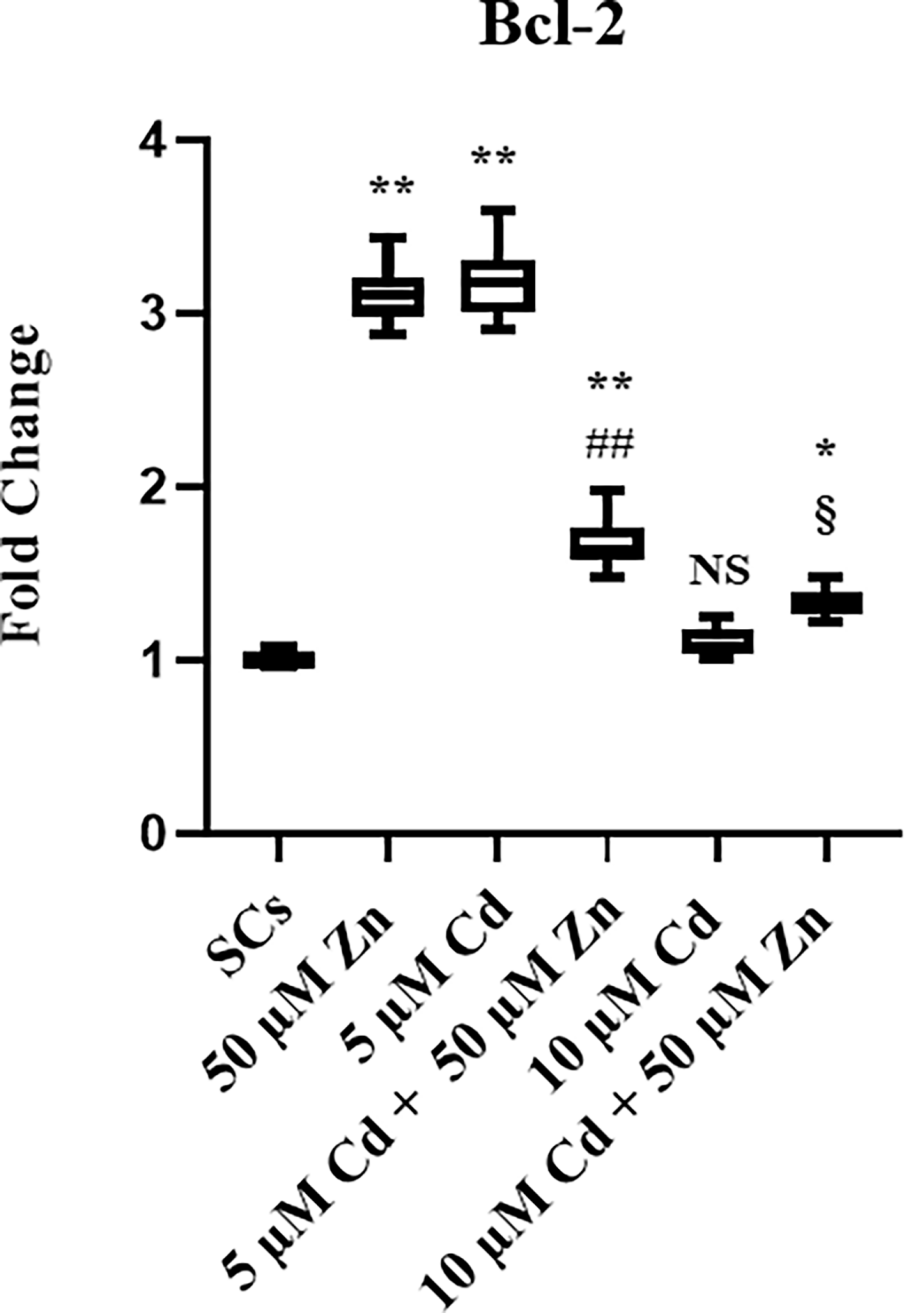
Real-Time PCR analysis of Bcl-2. Gene expression of Bcl-2 in the absence (unexposed-control group, SCs) or presence of 5 or 10 µM CdCl2 and/or 50 µM ZnCl2 for 48 h. Data represent the mean ± S.E.M. (Not significant, NS). NS vs SCs, *p < 0.05 and **p < 0.001, ##p < 0.001 vs 5 µM Cd, §p < 0.05 vs 10 µM of three independent experiments, each performed in triplicate.

**Figure 5A** shows that AMH gene expression was not influenced by Zn exposure, while it was significantly downregulated in Cd-exposed SCs (**p < 0.001 with −21% and −79% at 5 and 10 μM Cd concentrations, respectively) compared with unexposed controls. Zn and Cd co-treatment showed different results: 50 μM Zn in combination with 5 μM Cd upregulated AMH gene expression in SCs (##*p* < 0.001 with +34% with respect to SCs exposed to 5 μM Cd alone) up to the levels of untreated controls; 50 μM Zn in combination with 10 μM Cd did not modify AMH gene expression with respect to 10 μM Cd alone.

**Figure 5B** shows that inhibin B gene expression was not influenced by Zn exposure, while it was significantly downregulated in Cd-exposed SCs (**p < 0.001 with −55% and −88% at 5 and 10 μM Cd concentrations, respectively) compared with unexposed controls. Instead, co-treatment with Zn in combination with both Cd concentrations upregulated inhibin B gene expression in SCs (##*p* < 0.001 with +39.5% at 5 μM Cd + 50 μM Zn and §§*p* < 0.001 with +86.6% at 10 μM Cd + 50 μM Zn, respectively), compared with SCs exposed to 5 and 10 μM Cd alone.

Compared with unexposed SC controls, the AMH secretion was not influenced by Zn (**Figure 5C**), while it was significantly decreased after Cd exposure (**Figure 5C**). Differences in the AMH protein secretion were −42.1% and −83.6% at 5 and 10 μM Cd concentrations, respectively (**p < 0.001) in comparison with the unexposed SCs; after Zn and Cd co-treatment, the AMH protein secretion was increased in 5 μM Cd co-treated SCs (+35.9%; #*p* < 0.001 *vs*. SCs exposed to 5 μM Cd alone) but was unaffected in 10 μM Cd co-treated SCs *vs*. SCs exposed to 10 μM Cd alone.

Compared with unexposed SC controls, the inhibin B secretion was not influenced by Zn, while it was significantly decreased after Cd exposure (**Figure 5D**) with −51.4% and −88.2% at 5 and 10 μM Cd concentrations, respectively (**p < 0.001); Zn and Cd co-treatment increased inhibin B protein secretion in the 5 μM Cd co-treated SCs (+28.7%; **p < 0.001 *vs*. unexposed SCs; and +165%; ##*p* < 0.001 *vs*. SCs exposed to 5 μM Cd alone) but was unaffected in 10 μM Cd co-treated SCs *vs*. SCs exposed to 10 μM Cd alone.

**Figure 6:**
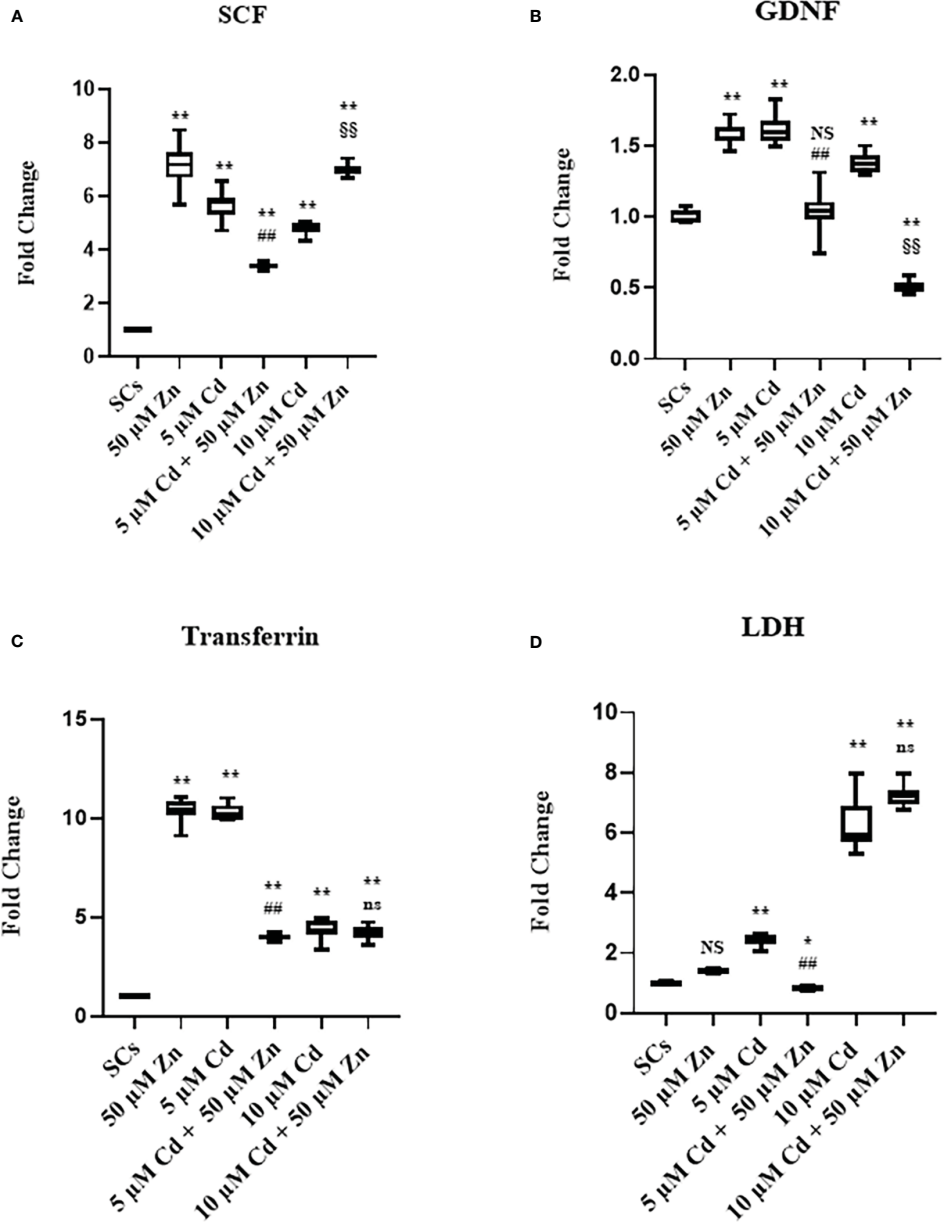
Real-time PCR analysis of SC functionality. Gene expression of SCF **(A)** GDNF **(B)**, transferrin **(C)**, and lactate dehydrogenase (LDH) **(D)** in the absence (unexposed-control group, SCs) or presence of 5 or 10 µM CdCl2 and/or 50 µM ZnCl2 for 48 h. Data presented as the mean ± SEM. (Not significant, NS). NS vs. SCs, *p < 0.05 and **p < 0.001, #p < 0.05 and ##p < 0.001 vs. 5 µM Cd, §p < 0.05 and §§ p < 0.001 vs. 10 μM. (Not significant, ns) ns vs. 10 μM of three independent experiments, each performed in triplicate.

The gene expression of SCF, GDNF, transferrin, and LDHA was upregulated at each experimental condition in comparison with unexposed SCs controls (**p < 0.001 *vs*. unexposed SCs; ##*p* < 0.001 *vs*. SCs exposed to 5 μM Cd alone; §§*p* < 0.001 *vs*. SCs exposed 10 μM Cd + 50 μM Zn, respectively) compared with unexposed controls (**Figures 6A–D**).

**Figure 7:**
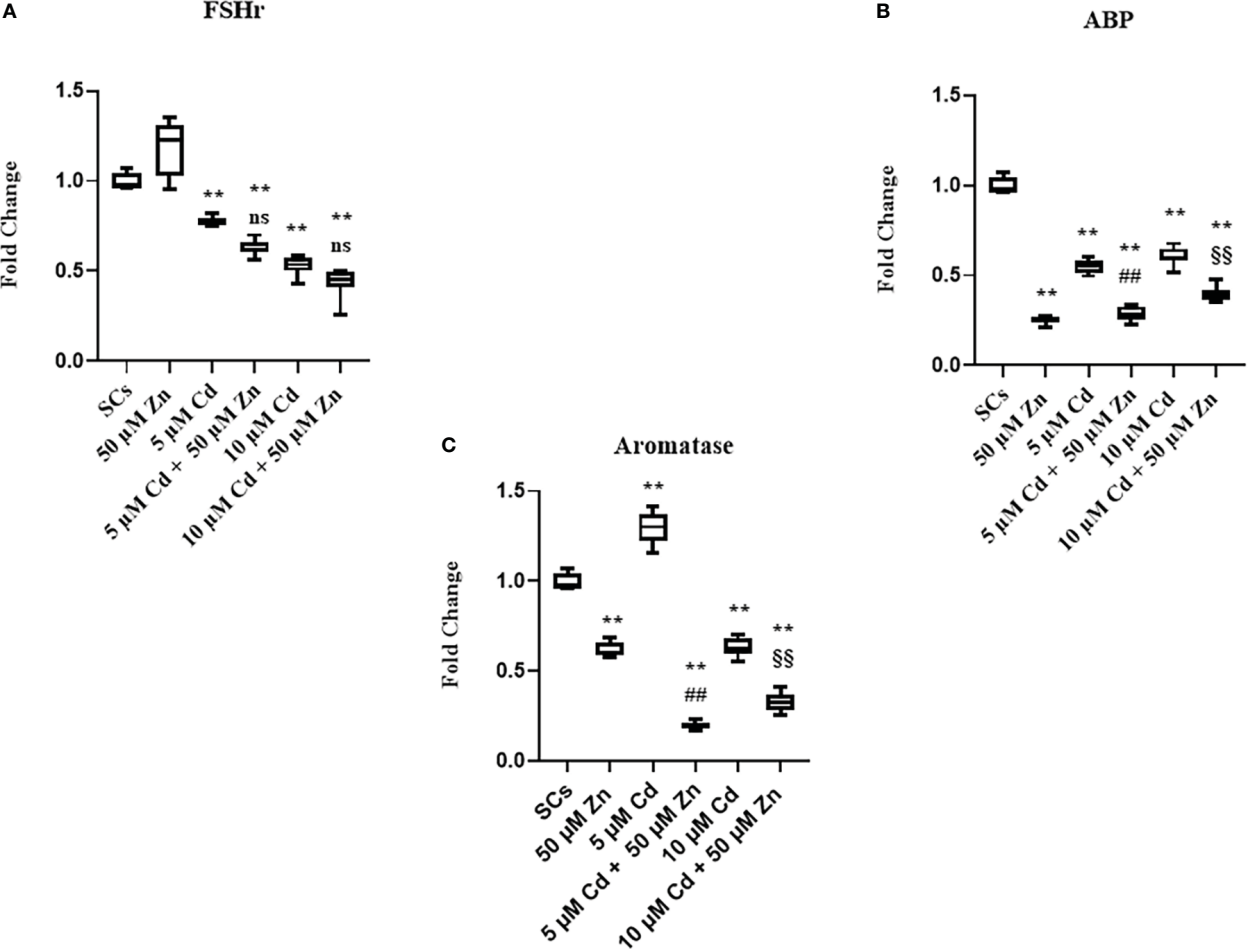
Real-time PCR analysis of SC functionality. Gene expression of FSHr **(A)**, androgen binding protein (ABP) **(B)**, and aromatase **(C)** in the absence (unexposed-control group, SCs) or presence of 5 or 10 μM CdCl2 and/or 50 µM ZnCl2 for 48 h. Data presented as the mean ± SEM. (Not significant, NS). NS vs. SCs, *p < 0.05 and **p < 0.001, #p < 0.05 and ##p< 0.001 vs. 5 µM Cd, §p<0.05, §§p< 0.001 vs. 10 µM, (not significant, ns) ns vs. 10 µM of three independent experiments, each performed in triplicate.

Moreover, we observed downregulation of FSH-r, ABP, and aromatase gene expression at each experimental condition (**p < 0.001 *vs*. unexposed SCs; ##*p* < 0.001 *vs*. SCs exposed to 5 μM Cd alone; §§*p* < 0.001 *vs*. SCs exposed 10 μM Cd + 50 μM Zn, respectively) compared with unexposed controls (**Figures 7A–C**).

These data showed in the tested experimental conditions that SCs were induced to produce factors necessary for differentiation and sustenance of spermatogonial cells as a response to a noxious stimulus. Furthermore, the data showed that SCs lost the ability to concentrate and transform androgens, activities that Zn treatment was unable to restore (52–54).

### 3.4 Antioxidant response: Nrf2, SOD1, GSHPx, and HO-1 gene expressions and intracellular ROS production

The results demonstrate that Zn co-treatment, in order to alleviate the oxidative damage, triggered antioxidant response in SCs by promoting the nuclear factor erythroid 2-related factor (Nrf2) pathway, but was able to counteract the oxidative stress only at subtoxic Cd concentration.

To investigate whether, in SCs, metal ions alone and in co-treatment activated Nrf2 and its downstream phase II detoxification genes, Nrf2, superoxide dismutase-1 (SOD1), glutathione peroxidase (GSHPx), and heme oxygenase (HO-1) gene expressions were evaluated.

**Figure 8:**
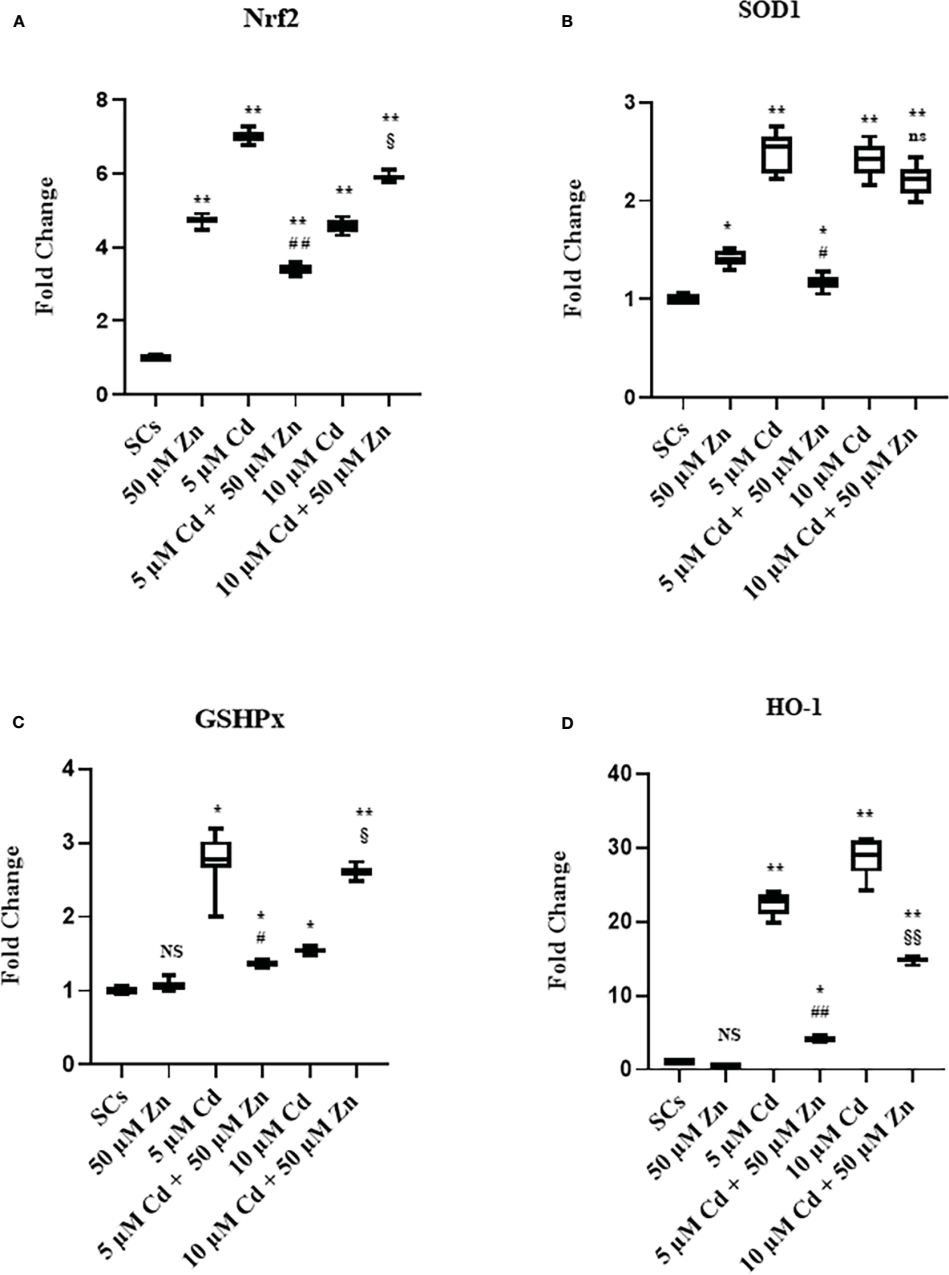
Real-time PCR analysis of antioxidant enzymes. Gene expression of Nrf2 **(A)**, SOD1 **(B)**, GSHPx **(C)**, and HO-1 **(D)** in the absence (unexposed-control group, SCs) or presence of 5 or 10 µM CdCl_2_ and/or 50 µM ZnCl2 for 48 h. Data presented as the mean ± SEM (NS *vs*. SCs, *p < 0.05 and **p < 0.001, #p < 0.05 and ##*p* < 0.001 vs. 5 µM Cd, §p < 0.05 and §§p < 0.001, (not significant, ns), ns vs. 10 µM of three independent experiments, each performed in triplicate.

**Figure 8A** shows that exposure of SCs to Zn alone upregulated Nrf2 gene expression by 370%, compared with unexposed SCs controls (**Figure 8A**, **p < 0.001 *vs*. unexposed SCs, set as 1 in the graphs).

Exposure to 5 and 10 μM Cd concentrations increased the Nrf2 gene expression by 600% and 360%, respectively, compared with unexposed SC controls (**Figure 8B**, **p < 0.001 *vs*. unexposed SCs, set as 1 in the graphs).

Nrf2 gene expression was increased in Zn co-treatment with 5 μM Cd by approximately 241% with respect to the unexposed SCs (**p < 0.001) but was decreased by 51% in comparison with 5 μM Cd-treated SCs alone (##*p* < 0.001).

Meanwhile, co-treatment with 10 μM Cd + 50 μM Zn increased Nrf2 gene expression (§*p* < 0.05) in comparison with 10 μM Cd alone.

**Figure 8B** shows that exposure of SCs to Zn alone upregulated SOD1 gene expression by 41%, compared with unexposed SC controls (*p < 0.05 *vs*. unexposed SCs, set as 1 in the graphs). Instead, Zn alone did not change GSHPx (**Figure 8C**) and HO-1 gene expressions compared with unexposed SC controls (**Figure 8D**).

Exposure of SCs to both Cd concentrations increased the gene expression of SOD1, GSHPx, and HO-1 (**Figures 8B–D**, *p < 0.05 and **p < 0.001 *vs*. unexposed SCs, set as 1 in the graphs). Co-treatment with 50 μM Zn significantly reduced all the aforementioned gene expressions in 5 μM Cd co-treated SCs compared with SCs exposed to 5 μM Cd alone (**Figures 8B–D**, *p < 0.05 *vs*. SCs exposed to 5 μM Cd alone), but showed different effects in 10 μM Cd co-treated SCs. In particular, co-treatment had no effect on SOD1, upregulated GSHPx by approximately 69%, and downregulated HO-1 gene expressions by 48% (**Figures 8B–D**, §*p* < 0.05 and §§*p* < 0.001 *vs*. SCs treated with 10 μM Cd alone).

**Figure 9:**
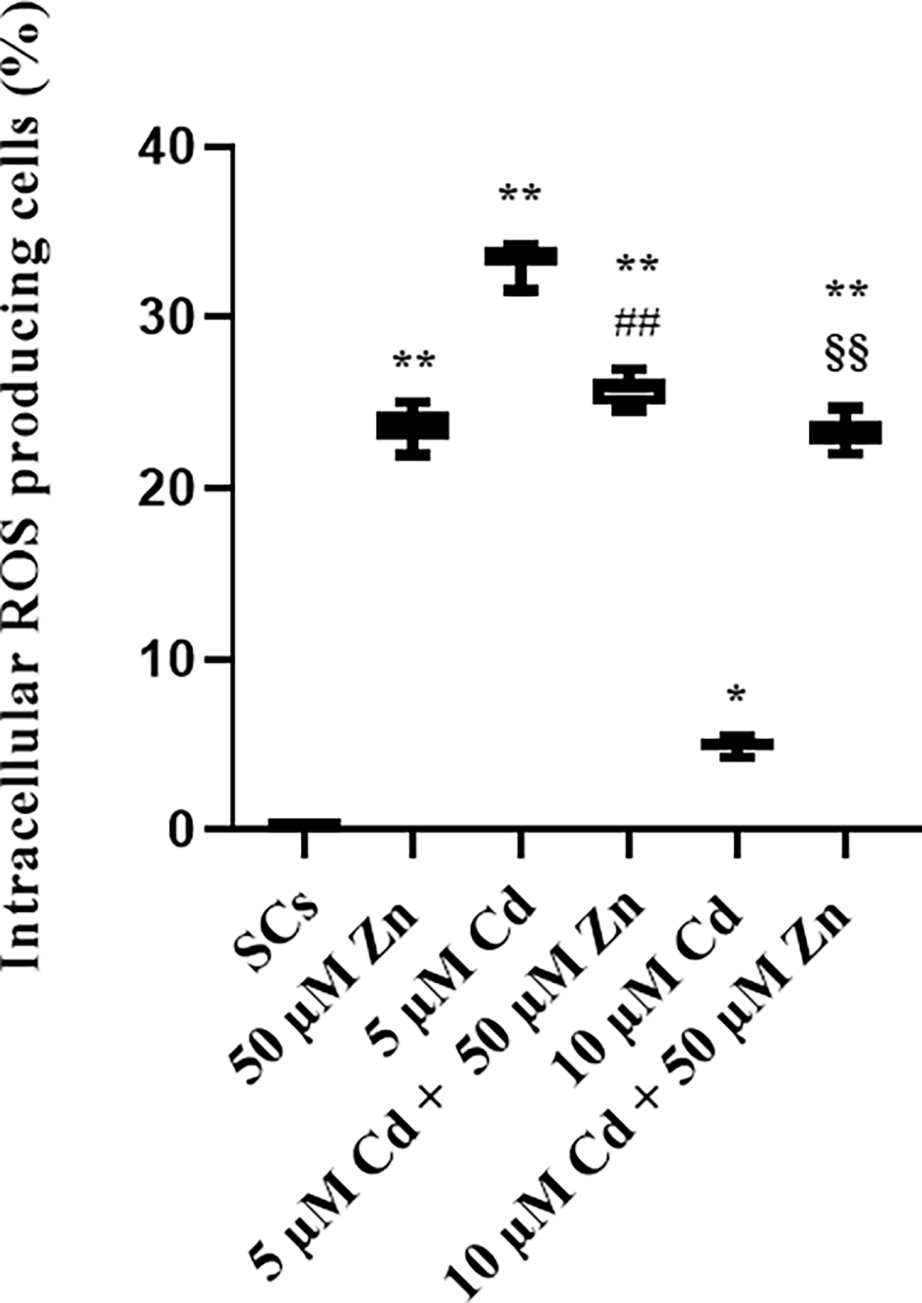
ROS assay. Intracellular ROS-producing cells in the absence (unexposed-control group, SCs) or presence of 5 or 10 µM CdCl_2_ and/or 50 µM ZnCl_2_ for 48 h. Data presented as the mean ± SEM. (*p < 0.05 and **p < 0.001, ##*p* < 0.001 *vs*. 5 µM Cd, §§*p* < 0.001 *vs*. 10 µM of three independent experiments, each performed in triplicate).

We evaluated the production of ROS as the most important intracellular effects elicited by Cd exposure and then influenced by Zn-Cd co-treatment, in terms of percentage of ROS-producing cells. **Figure 9** shows a significant increase in ROS-producing cells after Zn treatment and 5 and 10 μM Cd alone exposure (+23.81% and +40.9% with **p < 0.001 *vs*. unexposed SCs, and +4.9% with *p < 0.05 *vs*. unexposed SCs, respectively, set as 0 in the graphs). Zn (50 µM) and Cd co-treatment showed different results: in combination with 5 μM Cd, the percentage of ROS-producing cells was decreased by 23% with respect to 5 μM Cd alone (##*p* < 0.001), while in combination with 10 μM Cd, it increased by approximately 382% compared with SCs exposed to 10 μM Cd alone (§§*p* < 0.001).

### 3.5 WB analysis of ERK 1-2 and AKT signaling pathways

Our data demonstrated that the co-treatment with the oligoelement increased the upregulation of the extracellular signal-regulated kinase 1/2 (ERK 1/2) signal pathway and was therefore responsible for the attempt to counteract the negative effects of the heavy metal. On the contrary, the upregulation of the anti-apoptotic pathway of serine/threonine protein kinase (AKT) exhibited a direct dose-response increase with the concentration of Cd in co-treatment with Zn.

**Figure 10:**
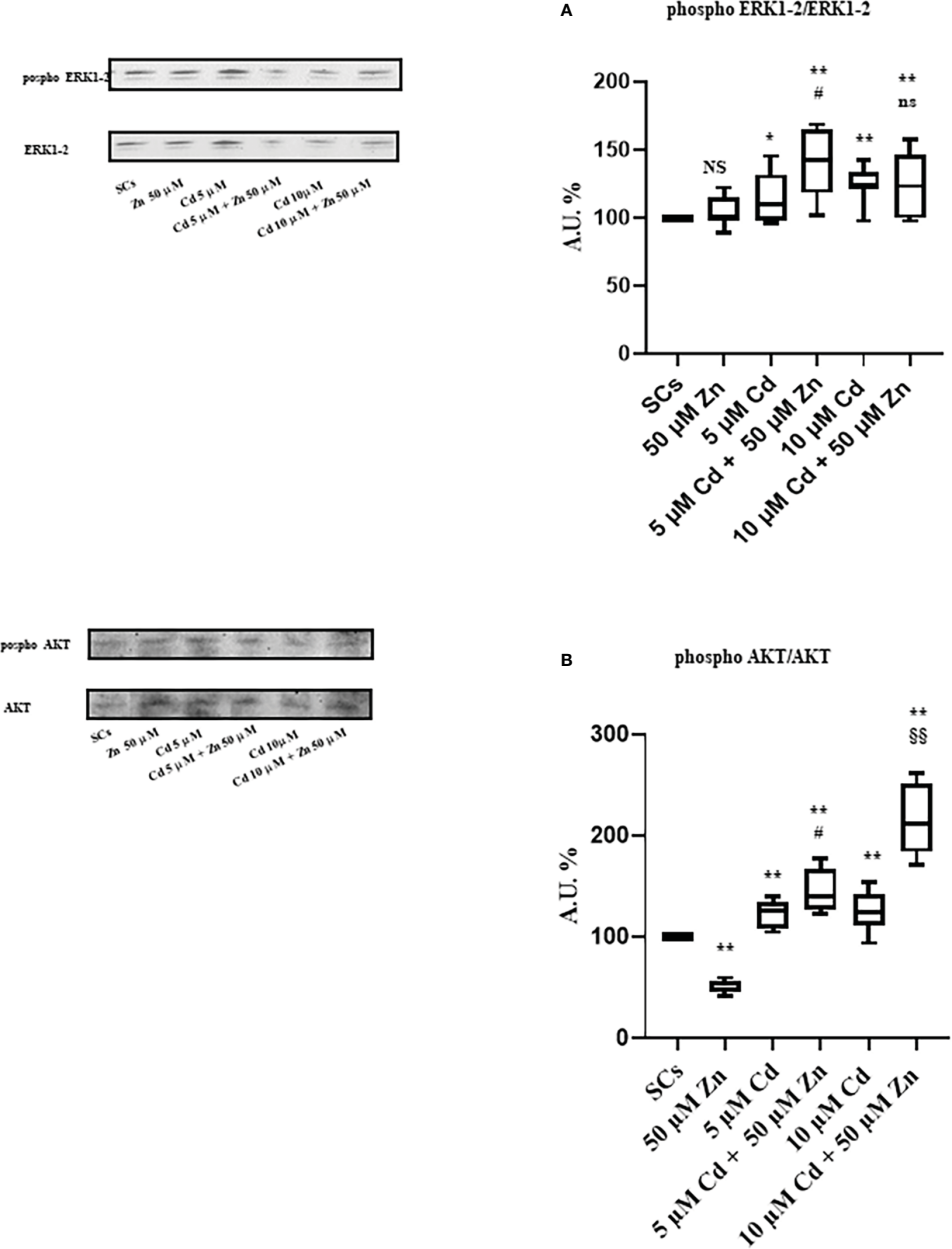
WB analysis of ERK1/2 and AKT pathways. **(A)** Immunoblots (upper left) and densitometric analysis (upper right) of the phosphorylation ratio of ERK1/2 in the absence (unexposed-control group, SCs) or presence of 5 or 10 µM CdCl_2_ and/or 50 μM ZnCl_2_ for 48 h. **(B)** Immunoblots (lower left) and densitometric analysis (lower right) of phosphorylation ratio of AKT in the absence (unexposed-control group, SCs) or presence of 5 or 10 µM CdCl_2_ and/or 50 µM ZnCl_2_ for 48 h. Data presented as the mean ± SEM (NS *vs*. SCs, *p < 0.05 and **p < 0.001 *vs*. SCs, #*p* < 0.05 *vs*. 5 µM Cd, §§*p* < 0.001 *vs*. 10 µM, ns *vs*. 10 μM of three independent experiments, each performed in triplicate) [*p < 0.05 and **p < 0.001, with respect to unexposed SCs (black dotted line) of three independent experiments, each performed in triplicate]. A.U., arbitrary units.

WB analysis demonstrated that Zn alone did not influence the ERK1/2 phosphorylation ratio compared with unexposed SCs, while 5 μM and 10 μM Cd increased it by approximately 15% and 25% (**Figure 10A**; *p < 0.05 and **p < 0.001 *vs*. unexposed SCs). Zn co-treatment with 5 μM Cd increased ERK 1-2 phosphorylation ratio by 40% *vs*. unexposed SCs and by 22% *vs*. SCs exposed to 5 μM Cd alone (**Figure 10A**; **p < 0.001 and #*p* < 0.05, respectively). Zn co-treatment with 10 μM Cd increased ERK 1-2 phosphorylation ratio by 25% *vs*. unexposed SCs (**Figure 10A**; **p < 0.001) but did not change in comparison with SCs exposed to 10 μM Cd alone.

Conversely, WB analysis reported in **Figure 8B** shows that Zn alone decreased AKT phosphorylation ratio by approximately 49%, while it increased at 5 μM and 10 μM Cd concentrations by approximately 21% and 25%, respectively (*p < 0.05, and **p < 0.001 *vs*. unexposed SCs). **Figure 10B** shows that Zn co-treatment with 5 μM Cd increased AKT phosphorylation ratio by 44% *vs*. unexposed SCs and by 19% *vs*. SCs exposed to 5 μM Cd alone (**Figure 10A**; **p < 0.001 and #*p* < 0.05, respectively). Zn co-treatment with 10 μM Cd increased AKT phosphorylation ratio by 112% *vs*. unexposed SCs (**Figure 10A**; **p < 0.001) and by 70% *vs*. SCs exposed to 10 μM Cd alone (**Figure 10A**; §§ *p* < 0.001).

## 4 Discussion

The present study was performed in order to evaluate whether the altered function and the cytotoxic effects in an *in vitro* model of porcine pre-pubertal SCs exposed to an acute stimulus with Cd, as previously reported (30), could be counteracted by the co-treatment with an oligoelement such as Zn.

Although *in vitro* studies do not represent a full realistic model of how substances will interact with the specific organ of the body *in vivo*, primary SC cultures have been recognized as a valuable *in vitro* system to study the effects of heavy metals and toxic substances (28–30), to test the action of drugs (55) and the protective effect of antioxidant substances under heavy metal exposure (56). Since our model is of a superior mammal, whose physiology is very similar to humans, our results pave the way for studying toxicology and therapeutic regimen in our species.

Cd is a heavy metal found as a contaminant in the environment, especially in food (57), and is classified as a highly toxic element associated with male infertility (58).

Indeed, one of the target organs of the Cd is the testis, where this metal can accumulate after exposure even during fetal and pre-pubertal life, thus altering the seminiferous tubules, SCs, blood–testis barrier, and Leydig cells (18).

Zn is a heavy metal as well, but shows a physiological function in normal spermatogenesis. Zn may have preventive therapeutic effects and/or may improve testicular function after toxicosis from metals such as Cu (59). Moreover, in an *in vivo* rat model in which Cd exposure induced oxidative stress in the testis, Cd + Zn co-exposure reversed the impaired testicular function (60).

Kheradmand et al. also reported the protective *in vitro* effect of Zn pre-treatment of TM4 mouse SCs against Cd toxicity (61) due to Zn-dependent increased gene expression of metallothioneins (MTs), heavy metal-binding proteins with a fundamental role in the homeostatic control and detoxification of Cd.

To the best of our knowledge, there are only a few studies concerning Cd toxicity during prepubertal life, since most of the *in vivo* and *in vitro* studies on this argument have been performed on adult animal and cellular models, mainly murine.

Therefore, considering that our well-established model of primary SC cultures from pre-pubertal pig testes is an important tool to characterize testicular toxicity, we employed SCs to examine the damage undergone by SCs exposed to subtoxic (5 μM) and toxic (10 μM) levels of Cd for 48 h in terms of metabolic and functional cell activity, cell cycle and apoptosis, and signaling pathways associated with oxidative stress, intracellular ROS production, and cell survival. Concurrently, we investigated whether co-treatment with 50 μM Zn could counteract and, eventually, restore the Cd-altered parameters.

Cd concentrations were chosen using data from the literature (30, 62). A 50 μM Zn (pharmacologic dosage) concentration was chosen based on literature data and confirmed by our preliminary cytotoxicity assays using 3-(4,5-dimethylthiazol-2-yl)-2,5-diphenyl-2H-tetrazolium bromide (MTT) (40).

The cytotoxic effect of Cd is already known (30, 58) and was also confirmed in our study by MTT analysis, which revealed a significant dose-dependent decrease of metabolically active cells after Cd exposure.

Zn alone exposure resulted in a slight nonsignificant decrease in metabolically active SCs, whereas co-treatment with 50 μM Zn at concentrations of 5 μM or 10 μM Cd increased metabolically active SCs, thereby restoring or markedly attenuating, respectively, the cytotoxic effects of Cd.

When cell cycle was analyzed, we found that Cd strongly increased the percentage of cells going into the sub-G_1_ area in a dose-dependent way and decreased SCs in the G_0_/G_1_ phase, while the proportion of cells in the S phase remained stable.

The results obtained in our SC system confirmed that Cd was responsible for halting the cell cycle, blocking proliferation, and increasing cell death, probably due to Cd damage to DNA, as already shown in a model of SC piglet (26).

Our study demonstrated that co-treatment of Zn with either Cd concentration was not able to resume cell cycle progression to the levels of untreated controls.

When we deepened the effects exerted by Cd exposure on cell death, we found a dose-dependent pro-apoptotic and necrotic effect in accordance with previous findings (27, 30).

However, when we added Zn and Cd to cell cultures at the same time, we were somewhat surprised to observe that the percentage of apoptotic SCs was significantly increased after co-treatment with both 5 and 10 µM Cd concentration.

It is already known that Zn alters cell proliferation and apoptosis with complex and controversial effects depending on cell type (63, 64). Moreover, it has been shown that Zn can have conflicting and biphasic (induction and inhibition) effects on apoptosis depending on its concentration (65). Indeed, in other cell systems such as PC12 cells, higher concentrations, from 100 to 500 µM Zn, have been shown to synergistically promote Cd-induced apoptosis (66), suggesting that our SCs are more sensitive to Zn and Cd co-treatment than other cell models. To the best of our knowledge, this is the first time that the pro-apoptotic effect of Zn/Cd co-treatment reported in the present work has been demonstrated in porcine pre-pubertal SCs.

Among proteins that regulate apoptosis, Caspase-3 is a key mediator of programmed cell death and is associated with the initiation, execution, and completion of apoptosis (47–51). When we assayed the protein expression, we found a downregulation of an inactive 35-kDa form and active fragments after Cd exposure alone or in combination with Zn, compared with untreated SCs. This is in contrast with the results of apoptosis and is probably due to the progress toward the final degradation phase of apoptosis (52).

Among the proteins that govern apoptosis, the Bcl-2 family of proteins constitutes a complex network of molecular sentinels, some of which promote cell survival (e.g., Bcl-2, among others) or induce apoptosis (i.e., BAX gene).

In some cell types, such as prostate cancer cells, Zn has been seen to induce mitochondrial apoptogenesis associated with an increase in the cellular level of Bax (67), whereas either cellular or mitochondrial Bcl-2 was stalled, such that the Bax/Bcl-2 ratio is increased, which is a pro-apoptotic condition.

Treatment with Zn alone and subtoxic Cd produced an upregulation of Bcl-2 gene expression probably as a defense mechanism implemented by SCs in anti-apoptotic function. At the toxic concentration of Cd, Bcl-2 gene expression was not different from untreated control as though the cell was no longer able to activate the defense mechanism toward the metal, thus leading to increased apoptosis.

The interesting fact is that with subtoxic Cd/Zn co-treatment, the gene expression of Bcl-2 was reduced and apoptosis was increased. Thus, the regulatory effect of Zn on Bcl-2 expression appears to parallel the synergistic action of Zn and Cd in promoting apoptosis of SCs.

The effects of Cd on SCs functionality were evaluated by studying the gene expression and protein secretion levels of AMH and inhibin B. AMH is a protein belonging to the TGF-beta family; as it is exclusively secreted by SCs, it is considered a useful marker of testicular function during the male prepubertal period (68). Another specific and significant marker of SC function is inhibin B, a protein that exerts a negative feedback on FSH secretion and is clinically useful to estimate the presence and function of SCs during infancy (30). In our study, we found that Zn alone did not change AMH and inhibin B mRNA and protein levels. Moreover, we found that the exposure to both Cd concentrations downregulated gene expression and protein secretion of AMH and inhibin B, confirming that Cd exposure impaired SC functionality, in agreement with previous reports on porcine prepubertal SCs (28, 30) or immature rat SCs (69) treated with various heavy metals.

Interestingly, co-treatment of Zn/5 µM Cd increased the gene expression and protein secretion of both Sertolian markers compared to Cd alone.

Instead, no effects were reported after co-treatment with Zn/10 µM Cd, hinting that the exposure to the Cd toxic concentration had such a big impact that Zn was unable to restore lost functionality.

Overall, these data would suggest that, although co-treatment at the subtoxic dose induced a pro-apoptotic effect, the cells that survived showed an improved functionality compared with Cd alone.

Interestingly, Cd exposure and/or co-treatment with Zn induced an increase in the gene expression of SCF, GDNF, transferrin, and LDH, in the absence of FSHr activation, by FSH, in agreement with what has been reported on cultures of rat SCs treated with cholera toxin and/or forskolin (53), mimicking what happens during prepubertal maturation of SCs during which their ability to respond to FSH increases and spermatogonial differentiation begins.

Cd exposure also induced a decrease in the gene expression of FSHr, ABP, and aromatase, demonstrating that the cells lose the ability to respond to the hormonal stimulus, to concentrate androgens, and consequently transform them, and that Zn alone or in co-treatment was unable to protect the cell from this specific alteration (54).

There is increasing evidence that the major effect of Cd on spermatogenesis is ROS induction in somatic and germ cells of the testis.

ROS are kept in a homeostatic balance between their production and their removal by an antioxidant system. If the balance is broken, oxidative stress occurs, which, by damaging DNA and enzymes, affects the functionality and/or viability of the cells involved in spermatogenesis (70–72).

SCs are equipped with antioxidant enzymes that provide protection against oxidative stress caused by potentially toxic ROS. In particular, the nuclear factor erythroid-2 related factor 2 (Nrf2) gene belonging to the Nrf2/antioxidant response element (Nrf2/ARE) signaling pathway encodes a crucial transcription factor in response to oxidative stress (73).

In fact, by triggering transcription of downstream target genes, Nrf2 induces the expressions of antioxidant and phase II detoxification genes in response to redox stress (74), among them superoxide dismutase-1 (SOD1), heme oxygenase I (HO-1), and glutathione peroxidase (GSHPx). SOD1 is an enzyme that contains both copper and Zn ions, catalyzes the dismutation of the superoxide radical (O2 −) into molecular oxygen (O2) or hydrogen peroxide (H2O2), acts as a major line of defense against oxygen-derived free radicals, and can be rapidly induced as a result of oxidative stress (75). GSHPx protects cell macromolecules from oxidative damage by catalyzing the reduction of hydrogen peroxide reaction and scavenging peroxides in the cell. HO-1 and their products protect against oxidative injury, regulate apoptosis, and modulate inflammation (70).

Given that several *in vitro* and *in vivo* studies support the hypothesis that Zn regulates the activity of Nrf2, thus mediating the expression of antioxidative proteins (76–79), we tested the effects of 50 µM Zn alone and/or in co-treatment with Cd on the antioxidant Nrf2 pathway and intracellular ROS production.

In our model system, we confirmed that treatment with 50 µM Zn alone induced an upregulated expression of Nrf2 gene, in parallel with an increased percentage of ROS-producing cells, suggesting that an increase in intracellular Zn may induce a transient elevation of oxidative stress as reported by others in different mammalian cells (80, 81). Among downstream genes, only SOD1 was upregulated, probably because it is the gene rapidly induced as the first line of defense against free radicals.

These results suggest that the oxidative stress condition was probably still kept under control by the cell, possibly by using its natural reservoir of reducing power such as glutathione (82).

This suggestion might also clarify why, in our system, Zn treatment alone did not affect the number of metabolically active cells and apoptosis.

Regarding Cd exposure, in our model, we found that subtoxic and toxic Cd exposure strongly activated the Nrf2 signaling pathway.

In particular, subtoxic and toxic Cd alone induced activation of the antioxidant pathway with upregulated gene expression of Nrf2 and its downstream genes SOD1, GSHPx, and HO-1, in order to counteract the oxidative stress demonstrated by the increased percentage of ROS-producing cells. Paradoxically, we could hypothesize that the lowest percentage of ROS-producing cells observed at the toxic dose could be explained by the increased number of dead cells.

Zn co-treatment with the subtoxic Cd concentration downregulated either the gene expression of Nrf2, its downstream genes, and the percentage of ROS-producing cells with respect to 5 µM Cd as expression of the improved detoxification activity. Instead, co-treatment with the toxic dose exhibited an increased Nrf2 and GSH-Px gene expression, with a concomitant increased percentage of ROS-producing cells, probably due to the improved cell viability exerted by Zn co-treatment, even though the normal condition has not been restored.

Overall, our results show that Zn and Cd act in different ways on the Nrf2 pathway and that regulation of Nrf2 and its downstream genes is a part of a complex and multifaceted relationship that needs to be further elucidated, and likely involves other cellular systems directly or indirectly linked to oxidative stress.

Among the cell signaling pathways affected by Cd exposure, we focused our attention on ERK 1-2, a member of the MAPK family, and PI3K/AKT signaling pathways (83) involved in survival cell response (84, 85).

In particular, the ERK 1-2/MAPK pathway is the major signaling pathway involved in cell growth and proliferation, where phosphorylation of ERK 1-2 represents the activation of the ERK 1-2/MAPK pathway. In the present work, we showed that Zn alone did not influence the ERK 1-2/MAPK pathway, while Cd affected the phosphorylation ratio by increasing it at both concentrations according to previously reported data (56).

Co-treatment with Zn in combination with both Cd concentrations induced a statistically significant upregulation of phosphorylation ratio compared with unexposed and Cd alone exposed SCs.

Such findings demonstrated the involvement of the ERK 1-2 pathway as target of metal ions and that Zn in combination with both Cd concentrations is able to activate this pathway in order to promote cell survival.

PI3K/AKT signaling favors cell-cycle progression (86) and provides protection from apoptosis, so that downregulation of AKT has been invoked to explain ROS-mediated apoptosis (87). We found that AKT phosphorylation, a well-known anti-apoptotic signal (28), was decreased after Zn alone exposure of SCs. On the contrary, we found an increase in AKT phosphorylation ratio at both subtoxic and toxic Cd concentrations, as an attempt to counteract cell apoptotic death as previously reported (56). Similarly, the co-treatment at both Cd concentrations upregulated the phosphorylation ratio in comparison with the metal alone, with respect to the unexposed SCs.

Zn is considered to be one of the most powerful stimulators of AKT (88), but in our system, such a stimulatory effect was only found when in combination with Cd.

In summary, to the best of our knowledge, the present work reports, for the first time, this trend in the ERK 1-2 and AKT pathways in SCs after co-treatment with Cd and Zn.

Taken together, our results show a complex picture, in which the protective effect of Zn, already widely known in the literature (54–57), also seems to be manifested in inducing apoptosis of SCs too severely damaged by Cd exposure to be rescued. SCs that actually survived the pro-apoptotic effect of co-administration of Zn with reduced dose of Cd indeed showed recovery of a portion of their functionality, shown by increased gene expression of the two major Sertolian markers, AMH and inhibin B.

Our hypothesis of this additional potential function of Zn is supported by data from the literature regarding its varied and biphasic role in the protection and induction of apoptosis in various cell lines (58–61) and in the toxicity expressed at high concentrations, which seem capable of damaging the tight junctions of SCs (58), a structure that is entirely essential for the proper conduct of spermatogenesis. It is important to note that the protective effect of Zn is believed not to be fully explained to date.

Its impact on the apoptotic selection on the cells based on their damage extent, coupled with Zn’s ability to induce the expression of metallothioneins genes, seems to be the current best explanation for our data, which overall show an interesting and somewhat unexpected effect on SC function.

## 5 Conclusions

The most striking result from the present study was that Zn/Cd co-treatment was able to overcome and restore the loss of functionality sustained by SCs exposed to subtoxic Cd concentration. According to the experimental results, our main hypothesis states that Zn/Cd co-treatment does not appear to have the power to restore Cd-induced damage in all cells but, by increasing apoptosis, it might be able to select SCs able to maintain or gain functionality close to untreated controls, thanks to the involvement of Nrf2, ERK1/2, and AKT pathways. This hypothesis requires further study to be confirmed, but together with the data already in the literature regarding the protective effects of Zn, it seems to adequately explain the complex and counterintuitive effects we observed on SCs.

Cd and Zn ions and redox signaling are intricately linked on multiple levels, and our model is useful to simulate the effects of early Cd damage on immature testis, effects that will certainly affect spermatogenesis in adults; in perspective, our model is suitable for further study aimed at evaluating the protective effects of Zn pre-treatment before Cd exposure.

The increasing exposure to multiple environmental pollutants, which seems difficult to reduce given their wide diffusion and the expanding use of some industrial processes, renders the search for potential protective agents essential and makes our study to evaluate the effect of Zn relevant.

Eventual encouraging results could suggest the application of Zn as a protective agent, suitable for prevention or therapy depending on the concentrations used, preventing the Cd-induced infertility in children and young adults.

## Data availability statement

The original contributions presented in the study are included in the article/**Supplementary Materials**. Further inquiries can be directed to the corresponding author.

## Ethics statement

Animal studies were conducted in agreement with the guidelines adopted by the Italian Approved Animal Welfare Assurance (A-3143-01) and the European Communities Council Directive of 24 November 1986 (86/609/EEC). The experimental protocols were approved by the University of Perugia.

## Author contributions

FM and IA designed the study, performed the experimental procedures, analyzed the data, and drafted and revised the manuscript. CB performed real-time PCR and analyzed the data. CL performed WB and analyzed the data. EE analyzed data and revised the manuscript. AMS and AP performed apoptosis, cell cycle, ROS assay, and analyzed the data. MCA performed ELISA assay and analyzed the data. FG and MC performed experiments. TB and GL gave experimental guidance, supervised, and revised the manuscript. All authors contributed to the article and approved the submitted version.

## Supplementary material

Supplementary Figure 1 Characterization of cultured SCs *in vitro* by fluorescence microscopy. **(A)** SCs after immunostaining with AMH and **(B)** vimentin antibodies and visualized by anti-goat Alexa Fluor 488 (green). **(C)** SCs after immunostaining with 3-βHSD antibody and visualized by anti-rabbit Alexa Fluor 488 (green). **(D)** SCs after immunostaining with ASMA antibody and visualized by anti-rabbit Alexa Fluor 488 (green). Nuclei are counterstained with DAPI (blue). The images are representative of three separate experiments.

## References

[1] Bhongade MB Prasad S Jiloha RC Ray PC Mohapatra S Koner BC. Effect of psychological stress on fertility hormones and seminal quality in male partners of infertile couples. Andrologia (2015) 47:336–42. doi: 10.1111/and.12268 24673246

[2] Ombelet W. WHO fact sheet on infertility gives hope to millions of infertile couples worldwide. Facts Views Vis Obgyn (2020) 12:249–51. PMC7863696 33575673

[3] Jungwirth A Giwercman A Tournaye H Diemer T Kopa Z Dohle G. European Association of urology guidelines on Male infertility: the 2012 update. Eur Urol (2012) 62:324–32. doi: 10.1016/j.eururo.2012.04.048 22591628

[4] Agarwal A Baskaran S Parekh N Cho CL Henkel R Vij S. Male Infertility. Lancet (2021) 397:319–33. doi: 10.1016/S0140-6736(20)32667-2 33308486

[5] Agarwal A Mulgund A Hamada A Chyatte MR. A unique view on male infertility around the globe. Reprod Biol Endocrinol (2015) 13:37. doi: 10.1186/s12958-015-0032-1 25928197 PMC4424520

[6] Hamada A Esteves SC Nizza M Agarwal A. Unexplained male infertility: diagnosis and management. Int Braz J Urol (2012) 38:576–94. doi: 10.1590/s1677-55382012000500002 23131516

[7] Ma Y He X Qi K Wang T Qi Y Cui L. Effects of environmental contaminants on fertility and reproductive health. J Environ Sci (China) (2019) 77:210–7. doi: 10.1016/j.jes.2018.07.015 30573085

[8] Bisht S Faiq M Tolahunase M Dada R. Oxidative stress and male infertility. Nat Rev Urol (2017) 14:470–85. doi: 10.1038/nrurol.2017.69 28508879

[9] Skakkebaek NE Rajpert-De Meyts E Main KM. Testicular dysgenesis syndrome: an increasingly common developmental disorder with environmental aspects. Hum Reprod (2001) 16:972–8. doi: 10.1093/humrep/16.5.972 11331648

[10] Skinner MK Bhandari RK Haque MM Nilsson EE. Environmentally induced epigenetic transgenerational inheritance of altered SRY genomic binding during gonadal sex determination. Environ Epigenet (2015) 1:dvv004. doi: 10.1093/eep/dvv004 27175298 PMC4862609

[11] Fayomi AP Orwig KE. Spermatogonial stem cells and spermatogenesis in mice, monkeys and men. Stem Cell Res (2018) 29:207–14. doi: 10.1016/j.scr.2018.04.009 PMC6010318 29730571

[12] Figà-Talamanca I Traina ME Urbani E. Occupational exposures to metals, solvents and pesticides: recent evidence on male reproductive effects and biological markers. Occup Med (Lond) (2001) 51:174–88. doi: 10.1093/occmed/51.3.174 11385122

[13] Balabanič D Rupnik M Klemenčič AK. Negative impact of endocrine-disrupting compounds on human reproductive health. Reprod Fertil Dev (2011) 23:403–16. doi: 10.1071/RD09300 21426858

[14] Ali H Khan E. What are heavy metals? long-standing controversy over the scientific use of the term ‘heavy metals’–proposal of a comprehensive definition. Toxicol Environ Chem (2018) 100:6–19. doi: 10.1080/02772248.2017.1413652

[15] Faroon O Ashizawa A Wright S Tucker P Jenkins K Ingerman L. Toxicological profile for cadmium. Atlanta (GA: Agency for Toxic Substances and Disease Registry (2012). 24049863

[16] Chirinos-Peinado DM Castro-Bedriñana JI. Lead and cadmium blood levels and transfer to milk in cattle reared in a mining area. Heliyon (2020) 6:e03579. doi: 10.1016/j.heliyon.2020.e03579 32195399 PMC7076556

[17] Khaneghah A Fakhri Y Nematollahi A Pirhadi M. Potentially toxic elements (PTEs) in cereal-based foods: A systematic review and meta-analysis. Trends Food Sci Techn. (2020) 96:30–44. doi: 10.1016/j.tifs.2019.12.007

[18] Wan HT Mruk DD Wong CK Cheng CY. The apical ES-BTB-BM functional axis is an emerging target for toxicant-induced infertility. Trends Mol Med (2013) 19:396–405. doi: 10.1016/j.molmed.2013.03.006 23643465 PMC3699959

[19] Kaur G Thompson LA Dufour JM. Sertoli cells–immunological sentinels of spermatogenesis. Semin Cell Dev Biol (2014) 30:36–44. doi: 10.1016/j.semcdb.2014.02.011 24603046 PMC4043859

[20] França LR Hess RA Dufour JM Hofmann MC Griswold MD Sertoli E. The sertoli cell: one hundred fifty years of beauty and plasticity. Andrology (2016) 4:189–212. doi: 10.1111/andr.12165 26846984 PMC5461925

[21] Guo J Nie X Giebler M Mlcochova H Wang Y Grow EJ. The dynamic transcriptional cell atlas of testis development during human puberty. Cell Stem Cell (2020) 26:262–276.e4. doi: 10.1016/j.stem.2019.12.005 31928944 PMC7298616

[22] Tan K Song HW Wilkinson MF. Single-cell RNAseq analysis of testicular germ and somatic cell development during the perinatal period. Development (2020) 147:dev183251. doi: 10.1242/dev.183251 31964773 PMC7033731

[23] Rebourcet D Wu J Cruickshanks L Smith SE Milne L Fernando A. Sertoli cells modulate testicular vascular network development, structure, and function to influence circulating testosterone concentrations in adult Male mice. Endocrinology (2016) 157:2479–88. doi: 10.1210/en.2016-1156 PMC4891787 27145015

[24] Unal E Yıldırım R Tekin S Demir V Onay H Haspolat YK. A novel mutation of AMHR2 in two siblings with persistent müllerian duct syndrom. J Clin Res Pediatr Endocrinol (2018) 10:387–90. doi: 10.4274/jcrpe.0013 PMC6280330 29687786

[25] Lindhardt Johansen M Hagen CP Johannsen TH Main KM Picard JY Jørgensen A. Anti-müllerian hormone and its clinical use in pediatrics with special emphasis on disorders of sex development. Int J Endocrinol (2013) 2013:198698. doi: 10.1155/2013/198698 24367377 PMC3866787

[26] Zhang M He Z Wen L Wu J Yuan L Lu Y. Cadmium suppresses the proliferation of piglet sertoli cells and causes their DNA damage, cell apoptosis and aberrant ultrastructure. Reprod Biol Endocrinol (2010) 8:97. doi: 10.1186/1477-7827-8-97 20712887 PMC3224921

[27] Yu X Hong S Faustman EM. Cadmium-induced activation of stress signaling pathways, disruption of ubiquitin-dependent protein degradation and apoptosis in primary rat sertoli cell-gonocyte cocultures. Toxicol Sci (2008) 104:385–96. doi: 10.1093/toxsci/kfn087 PMC2734295 18463101

[28] Mancuso F Arato I Lilli C Bellucci C Bodo M Calvitti M. Acute effects of lead on porcine neonatal sertoli cells *in vitro* . Toxicol In Vitro (2018) 48:45–52. doi: 10.1016/j.tiv.2017.12.013 29273543

[29] Marinucci L Balloni S Bellucci C Lilli C Stabile AM Calvitti M. Effects of nicotine on porcine pre-pupertal sertoli cells: An *in vitro* study. Toxicol In Vitro (2020) 67:104882. doi: 10.1016/j.tiv.2020.104882 32423882

[30] Luca G Lilli C Bellucci C Mancuso F Calvitti M Arato I. Toxicity of cadmium on sertoli cell functional competence: an *in vitro* study. J Biol Regul Homeost Agents (2013) 27:805–16. 24152845

[31] Hidiroglou M Knipfel JE. Zinc in mammalian sperm: a review. J Dairy Sci (1984) 67:1147–56. doi: 10.3168/jds.S0022-0302(84)81416-2 6378991

[32] Salgueiro MJ Weill R Zubillaga M Lysionek A Caro R Goldman C. Zinc deficiency and growth: current concepts in relationship to two important points: intellectual and sexual development. Biol Trace Elem Res (2004) 99:49–69. doi: 10.1385/bter:99:1-3:049 15235141

[33] Anjum MR Madhu P Reddy KP Reddy PS. The protective effects of zinc in lead-induced testicular and epididymal toxicity in wistar rats. Toxicol Ind Health (2017) 33:265–76. doi: 10.1177/0748233716637543 27102426

[34] Zhao J Dong X Hu X Long Z Wang L Liu Q. Zinc levels in seminal plasma and their correlation with male infertility: A systematic review and meta-analysis. Sci Rep (2016) 6:22386. doi: 10.1038/srep22386 26932683 PMC4773819

[35] Hartwig A. Zinc finger proteins as potential targets for toxic metal ions: differential effects on structure and function. Antioxid Redox Signal (2001) 3:625–34. doi: 10.1089/15230860152542970 11554449

[36] Sadik NA. Effects of diallyl sulfide and zinc on testicular steroidogenesis in cadmium-treated male rats. J Biochem Mol Toxicol (2008) 22:345–53. doi: 10.1002/jbt.20247 18972399

[37] Shahid MN Khan TM Neoh CF Lean QY Bukhsh A Karuppannan M. Effectiveness of pharmacological intervention among men with infertility: A systematic review and network meta-analysis. Front Pharmacol (2021) 16:638628. doi: 10.3389/fphar.2021.638628 PMC8415454 34483894

[38] Griswold MD. Sertoli Cell Biology, 2nd Edition, Editor: Griswold M, Elsevier (2014).

[39] Zhang L Guo M Liu Z Liu R Zheng Y Yu T. Single-cell RNA-seq analysis of testicular somatic cell development in pigs. J Genet Genomics (2022) 15:S1673–8527(22)00106-0. doi: 10.1016/j.jgg.2022.03.014 35436608

[40] Mancuso F Arato I Di Michele A Antognelli C Angelini L Bellucci C. Effects of titanium dioxide nanoparticles on porcine prepubertal sertoli cells: An "*In vitro*" study. Front Endocrinol (Lausanne). (2022) 3:751915. doi: 10.3389/fendo.2021.751915 PMC8762334 35046890

[41] Cannarella R Mancuso F Condorelli RA Arato I Mongioì LM Giacone F. Effects of GH and IGF1 on basal and FSH-modulated porcine sertoli cells in-vitro. J Clin Med (2019) 8(6):811. doi: 10.3390/jcm8060811 31174315 PMC6617362

[42] Duprez J Roma LP Close AF Jonas JC. Protective antioxidant and antiapoptotic effects of ZnCl2 in rat pancreatic islets cultured in low and high glucose concentrations. PloS One (2012) 7(10):e46831. doi: 10.1371/journal.pone.0046831 23056475 PMC3463538

[43] Castellini C Mattioli S Dal Bosco A Collodel G Pistilli A Stabile AM. *In vitro* effect of nerve growth factor on the main traits of rabbit sperm. Reprod Biol Endocrinol (2019) 17(1):93. doi: 10.1186/s12958-019-0533-4 31718673 PMC6849245

[44] Arcidiacono P Ragonese F Stabile A Pistilli A Kuligina E Rende M. Antitumor activity and expression profiles of genes induced by sulforaphane in human melanoma cells. Eur J Nutr (2018) 57(7):2547–69. doi: 10.1007/s00394-017-1527-7 PMC6182666 28864908

[45] Bassani B Bartolini D Pagani A Principi E Zollo M Noonan DM. Fenretinide (4-HPR) targets caspase-9, ERK 1/2 and the Wnt3a/β-catenin pathway in medulloblastoma cells and medulloblastoma cell spheroids. PloS One (2016) 11(7):e0154111. doi: 10.1371/journal.pone.0154111 27367907 PMC4930187

[46] Hong F Zhao X Chen M Zhou Y Ze Y Wang L. TiO2 nanoparticles-induced apoptosis of primary cultured sertoli cells of mice. J BioMed Mater Res A. (2016) 104(1):124–35. doi: 10.1002/jbm.a.35548 26238530

[47] Antognelli C Mancuso F Frosini R Arato I Calvitti M Calafiore R. Testosterone and follicle stimulating hormone-dependent glyoxalase 1 up-regulation sustains the viability of porcine sertoli cells through the control of hydroimidazolone- and argpyrimidine-mediated NF-κB pathway. Am J Pathol (2018) 188(11):2553–63. doi: 10.1016/j.ajpath.2018.07.013 30125541

[48] Cannarella R Arato I Condorelli RA Luca G Barbagallo F Alamo A. The IGF1 receptor is involved in follicle-stimulating hormone signaling in porcine neonatal sertoli cells. J Clin Med (2019) 8(5):577. doi: 10.3390/jcm8050577 31035547 PMC6571966

[49] Bradford MM. A rapid and sensitive method for the quantitation of microgram quantities of protein utilizing the principle of protein-dye binding. Anal Biochem (1976) 72:248–54. doi: 10.1016/0003-2697(76)90527-3 942051

[50] Cannarella R Arato I Condorelli RA Mongioì LM Lilli C Bellucci C. Effects of insulin on porcine neonatal sertoli cell responsiveness to FSH in vitro. J Clin Med (2019) 8(6):809. doi: 10.3390/jcm8060809 31174276 PMC6617126

[51] Fettucciari K Ponsini P Gioè D Macchioni L Palumbo C Antonelli E. Enteric glial cells are susceptible to clostridium difficile toxin b. Cell Mol Life Sci (2017) 74:1527–51. doi: 10.1007/s00018-016-2426-4 PMC11107567 27891552

[52] Valencia-Cruz G Shabala L Delgado-Enciso I Shabala S Bonales-Alatorre E Pottosin II. K(bg) and Kv1.3 channels mediate potassium efflux in the early phase of apoptosis in jurkat T lymphocytes. Am J Physiol Cell Physiol (2009) 297:C1544–53. doi: 10.1152/ajpcell.00064.2009 19794143

[53] Bhattacharya I Pradhan BS Sarda K Gautam M Basu S Majumdar SS. A switch in sertoli cell responsiveness to FSH may be responsible for robust onset of germ cell differentiation during prepubartal testicular maturation in rats. Am J Physiol Endocrinol Metab (2012) 303(7):E886–98. doi: 10.1152/ajpendo.00293.2012 22850685

[54] Majumdar SS Sarda K Bhattacharya I Plant TM. Insufficient androgen and FSH signaling may be responsible for the azoospermia of the infantile primate testes despite exposure to an adult-like hormonal milieu. Hum Reprod (2012) 27(8):2515–25. doi: 10.1093/humrep/des184 PMC3398678 22669085

[55] Arato I Ceccarelli V Mancuso F Bellucci C Lilli C Ferolla P. Effect of EPA on neonatal pig sertoli cells "*In vitro*": A possible treatment to help maintain fertility in pre-pubertal boys undergoing treatment with gonado-toxic therapies. Front Endocrinol (Lausanne). (2021) 12:694796. doi: 10.3389/fendo.2021.694796 34093450 PMC8174840

[56] Bartolini D Arato I Mancuso F Giustarini D Bellucci C Vacca C. Melatonin modulates Nrf2 activity to protect porcine pre-pubertal sertoli cells from the abnormal H2 O2 generation and reductive stress effects of cadmium. J Pineal Res (2022) 6:e12806. doi: 10.1111/jpi.12806 PMC9539639 35524288

[57] Satarug S. Dietary cadmium intake and its effects on kidneys. Toxics (2018) 6:E15. doi: 10.3390/toxics6010015 PMC5874788 29534455

[58] Zhu Q Li X Ge RS. Toxicological effects of cadmium on mammalian testis. Front Genet (2020) 11:527. doi: 10.3389/fgene.2020.00527 32528534 PMC7265816

[59] Kheirandish R Askari N Babaei H. Zinc therapy improves deleterious effects of chronic copper administration on mice testes: histopathological evaluation. Andrologia (2014) 46:80–5. doi: 10.1111/and.12047 23137167

[60] Amara S Abdelmelek H Garrel C Guiraud P Douki T Ravanat JL. Preventive effect of zinc against cadmium-induced oxidative stress in the rat testis. J Reprod Dev (2008) 54:129–34. doi: 10.1262/jrd.18110 17420618

[61] Kheradmand F Nourmohammadi I Modarressi MH Firoozrai M Ahmadi-Faghih MA. Differential gene-expression of metallothionein 1M and 1G in response to zinc in sertoli TM4 cells. Iran BioMed J (2010) 14:9–15. 20683493 PMC3878141

[62] Chang KL Hung TC Hsieh BS Chen YH Chen TF Cheng HL. Zinc at pharmacologic concentrations affects cytokine expression and induces apoptosis of human peripheral blood mononuclear cells. Nutrition (2006) 22(5):465–74. doi: 10.1016/j.nut.2005.11.009 16472982

[63] Bae SN Lee YS Kim MY Kim JD Park LO. Antiproliferative and apoptotic effects of zinc-citrate compound (CIZAR(R)) on human epithelial ovarian cancer cell line, OVCAR-3. Gynecol Oncol (2006) 103:127–36. doi: 10.1016/j.ygyno.2006.02.009 16624386

[64] Cuajungco MP Ramirez MS Tolmasky ME. Zinc: Multidimensional effects on living organisms. Biomedicines (2021) 9:208. doi: 10.3390/biomedicines9020208 33671781 PMC7926802

[65] Maret W. The redox biology of redox-inert zinc ions. Free Radic Biol Med (2019) 134:311–26. doi: 10.1016/j.freeradbiomed.2019.01.006 30625394

[66] Rahman MM Ukiana J Uson-Lopez R Sikder MT Saito T Kurasaki M. Cytotoxic effects of cadmium and zinc co-exposure in PC12 cells and the underlying mechanism. Chem Biol Interact (2017) 269:41–9. doi: 10.1016/j.cbi.2017.04.003 28390674

[67] Feng P Li T Guan Z Franklin RB Costello LC. The involvement of bax in zinc-induced mitochondrial apoptogenesis in malignant prostate cells. Mol Cancer (2008) 7:25. doi: 10.1186/1476-4598-7-25 18331646 PMC2329666

[68] Josso N Rey RA Picard JY. Anti-müllerian hormone: a valuable addition to the toolbox of the pediatric endocrinologist. Int J Endocrinol (2013) 2013:674105. doi: 10.1155/2013/674105 24382961 PMC3870610

[69] Monsees TK Franz M Gebhardt S Winterstein U Schill WB Hayatpour J. Sertoli cells as a target for reproductive hazards. Andrologia (2000) 32:239–46. doi: 10.1046/j.1439-0272.2000.00391.x 11021515

[70] Hassoun EA Stohs SJ. Cadmium-induced production of superoxide anion and nitric oxide, DNA single strand breaks and lactate dehydrogenase leakage in J774A. 1 Cell cultures. Toxicol (1996) 112:219–26. doi: 10.1016/0300-483x(96)03404-x 8845042

[71] Morielli T O'Flaherty C. Oxidative stress impairs function and increases redox protein modifications in human spermatozoa. Reproduction (2015) 149:113–23. doi: 10.1530/REP-14-0240 PMC5489333 25385721

[72] Wiwanitkit V. Cadmium related health effects. Environ Res (2012) 112:236. doi: 10.1016/j.envres.2011.12.005 22182981

[73] Yang SH Yu LH Li L Guo Y Zhang Y Long M. Protective mechanism of sulforaphane on cadmium-induced sertoli cell injury in mice testis *via* Nrf2/ARE signaling pathway. Molecules (2018) 23:E1774. doi: 10.3390/molecules23071774 PMC6100605 30029485

[74] Ma X He P Sun P Han P. Lipoic acid: An immunomodulator that attenuates glycinin-induced anaphylactic reactions in a rat model. J Agric Food Chem (2010) 58:5086–92. doi: 10.1021/jf904403u 20302377

[75] Loboda A Damulewicz M Pyza E Jozkowicz A Dulak J. Role of Nrf2/HO-1 system in development, oxidative stress response and diseases: an evolutionarily conserved mechanism. Cell Mol Life Sci (2016) 73:3221–47. doi: 10.1007/s00018-016-2223-0 PMC4967105 27100828

[76] Cortese MM Suschek CV Wetzel W Kröncke KD Kolb-Bachofen V. Zinc protects endothelial cells from hydrogen peroxide *via* Nrf2-dependent stimulation of glutathione biosynthesis. Free Radic Biol Med (2008) 44:2002–12. doi: 10.1016/j.freeradbiomed.2008.02.013 18355458

[77] Kaufman Z Salvador GA Liu X Oteiza PI. Zinc and the modulation of Nrf2 in human neuroblastoma cells. Free Radic Biol Med (2020) 155:1–9. doi: 10.1016/j.freeradbiomed.2020.05.010 32416241

[78] Maremanda KP Khan S Jena GB. Role of zinc supplementation in testicular and epididymal damages in diabetic rat: Involvement of Nrf2, SOD1, and GPX5. Biol Trace Elem Res (2016) 173:452–64. doi: 10.1007/s12011-016-0674-7 27025721

[79] Hübner C Haase H. Interactions of zinc- and redox-signaling pathways. Redox Biol (2021) 41:101916. doi: 10.1016/j.redox.2021.101916 33662875 PMC7937829

[80] Wiseman DA Sharma S Black SM. Elevated zinc induces endothelial apoptosis *via* disruption of glutathione metabolism: role of the ADP translocator. Biometals (2010) 23:19–30. doi: 10.1007/s10534-009-9263-y 19768661 PMC2904313

[81] Xue J Wang S Wu J Hannafon BN Ding WQ. Zinc at sub-cytotoxic concentrations induces heme oxygenase-1 expression in human cancer cells. Cell Physiol Biochem (2013) 32:100–10. doi: 10.1159/000350128 PMC3833100 23868099

[82] Adeoye O Olawumi J Opeyemi A Christiania O. Review on the role of glutathione on oxidative stress and infertility. JBRA Assist Reprod (2018) 22:61–6. doi: 10.5935/1518-0557.20180003 PMC5844662 29266896

[83] Renu K Chakraborty R Myakala H Koti R Famurewa AC Madhyastha H. Molecular mechanism of heavy metals (Lead, chromium, arsenic, mercury, nickel and cadmium) - induced hepatotoxicity - a review. Chemosphere (2021) 271:129735. doi: 10.1016/j.chemosphere.2021.129735 33736223

[84] Sun Y Liu WZ Liu T Feng X Yang N Zhou HF. Signaling pathway of MAPK/ERK in cell proliferation, differentiation, migration, senescence and apoptosis. J Recept Signal Transduct Res (2015) 35:600–4. doi: 10.3109/10799893.2015.1030412 26096166

[85] Taniguchi CM Emanuelli B Kahn CR. Critical nodes in signalling pathways: insights into insulin action. Nat Rev Mol Cell Biol (2006) 7:85–96. doi: 10.1038/nrm1837 16493415

[86] Abraham E. Akt/protein kinase b. Crit Care Med (2005) 33:S420–2. doi: 10.1097/01.ccm.0000191715.31970.d8 16340410

[87] Chetram MA Bethea DA Odero-Marah VA Don-Salu-Hewage AS Jones KJ Hinton CV. ROS-mediated activation of AKT induces apoptosis *via* pVHL in prostate cancer cells. Mol Cell Biochem (2013) 376:63–71. doi: 10.1007/s11010-012-1549-7 23315288 PMC3578043

[88] Eckers A Klotz LO. Heavy metal ion-induced insulin-mimetic signaling. Redox Rep. (2009) 14(4):141–6. doi: 10.1179/135100009X392610 19695120

