# Supplementary figures and images for: Zinc restores functionality in porcine prepubertal Sertoli cells exposed to subtoxic cadmium concentration via regulating the Nrf2 signaling pathway
Source: Front Endocrinol (Lausanne). 2023 Feb 10;14:962519. doi: 10.3389/fendo.2023.962519 (PMC9950629; doi:10.3389/fendo.2023.962519)

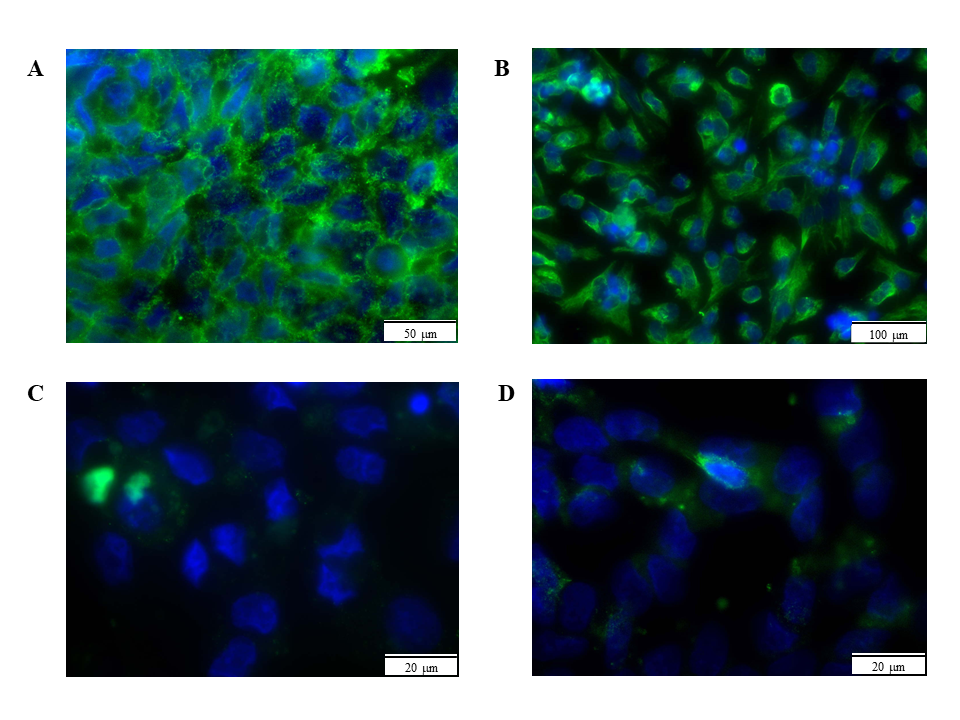

Supplement: Supplementary Figure 1 — Characterization of cultured SCs in vitro by fluorescence microscopy. (A) SCs after immunostaining with AMH and (B) vimentin antibodies and visualized by anti-goat Alexa Fluor 488 (green). (C) SCs after immunostaining with 3-βHSD antibody and visualized by anti-rabbit Alexa Fluor 488 (green). (D) SCs after immunostaining with ASMA antibody and visualized by anti-rabbit Alexa Fluor 488 (green). Nuclei are counterstained with DAPI (blue). The images are representative of three separate experiments. [file Image_1.tif]
